# Quantum Dot Biomimetic for SARS-CoV-2 to Interrogate Blood–Brain Barrier Damage Relevant to NeuroCOVID Brain Inflammation

**DOI:** 10.1021/acsanm.3c02719

**Published:** 2023-08-07

**Authors:** Wesley Chiang, Angela Stout, Francine Yanchik-Slade, Herman Li, Niccolò Terrando, Bradley L. Nilsson, Harris A. Gelbard, Todd D. Krauss

**Affiliations:** ^†^Department of Biochemistry and Biophysics, ^‡^Center for Neurotherapeutics Discovery and Department of Neurology, and ^§^Departments of Pediatrics, Neuroscience, and Microbiology and Immunology, University of Rochester Medical Center, Rochester, New York 14642, United States; ^∥^Department of Chemistry and ^⊥^The Institute of Optics, University of Rochester, Rochester, New York 14627, United States; # Department of Anesthesiology, Duke University Medical Center, Durham, North Carolina 27710, United States

**Keywords:** quantum dots, fluorescence, microscopy, neuroscience, inflammation, SARS-CoV-2, biomimetic

## Abstract

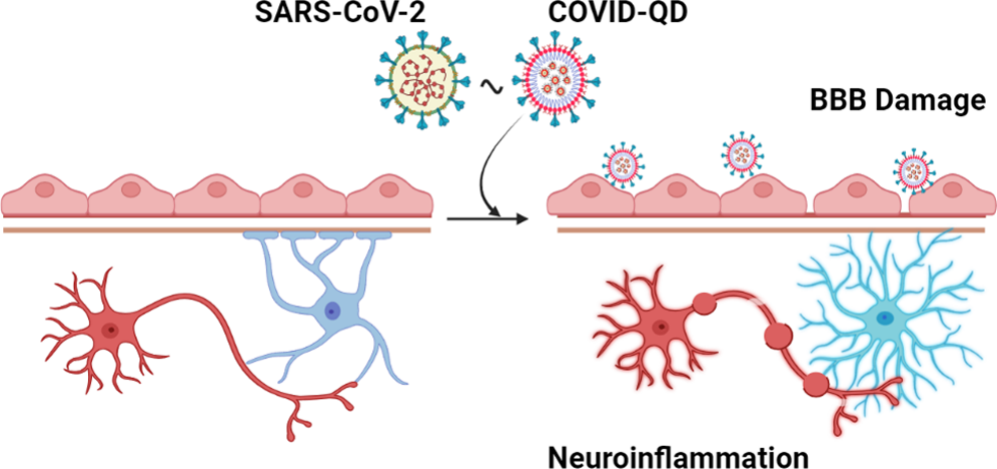

Despite limited evidence for infection of SARS-CoV-2 in the central nervous system, cognitive impairment is a common complication reported in “recovered” COVID-19 patients. Identification of the origins of these neurological impairments is essential to inform therapeutic designs against them. However, such studies are limited, in part, by the current status of high-fidelity probes to visually investigate the effects of SARS-CoV-2 on the system of blood vessels and nerve cells in the brain, called the neurovascular unit. Here, we report that nanocrystal quantum dot micelles decorated with spike protein (COVID-QDs) are able to interrogate neurological damage due to SARS-CoV-2. In a transwell co-culture model of the neurovascular unit, exposure of brain endothelial cells to COVID-QDs elicited an inflammatory response in neurons and astrocytes without direct interaction with the COVID-QDs. These results provide compelling evidence of an inflammatory response without direct exposure to SARS-CoV-2-like nanoparticles. Additionally, we found that pretreatment with a neuro-protective molecule prevented endothelial cell damage resulting in substantial neurological protection. These results will accelerate studies into the mechanisms by which SARS-CoV-2 mediates neurologic dysfunction.

## Introduction

“Long COVID” is an increasing medical concern that often consists of significant neurologic dysfunction, despite individuals having completely “recovered” from systemic COVID-19. Even with the numerous investigations that have been conducted relevant to COVID-19,^1^ the basis underlying the mechanisms of long COVID and possible neurodegeneration remain unclear. While studies of SARS-CoV-2 infection of nasal tissues as a direct route for neuroinvasion have demonstrated persistent infection of sustentacular cells in olfactory epithelium,^2,3^ the evidence for widespread infection of neurons, microglia, astrocytes (astroglia), and other neural cell types in the central nervous system (CNS) remains limited.^4^ Indeed, recent reports have suggested that acute and chronic inflammation in the periphery and CNS drives neurologic disease in long COVID.^5,6^ Current experimental and pathologic data suggests that CNS disease arises from dysregulation of the barrier-forming cells of the neurovascular unit (NVU).^7−9^ An important and unsolved question is whether disruption of the endothelium is sufficient to initiate neuroinflammation, or whether virus particles traverse the blood–brain barrier (BBB) and directly interact with cellular constituents of the CNS to induce a neuroinflammatory state. In theory, this question could be answered by directly visualizing whether SARS-CoV-2 virions are transported across the BBB into the CNS. However, simple and direct optical microscopic tools for imaging SARS-CoV-2 virions are lacking.

Fluorescently labeled virus-like nanoparticles (VLNPs) have the potential to clearly visualize whether SARS-CoV-2 virions are transported across the BBB into the CNS. Typically, VLNPs are either produced in cell expression vector systems to contain fluorescent proteins, or synthetically constructed with hydrophobic cores loaded with conventional fluorophores.^10−12^ Organic fluorophores, however, can be limited by relatively small absorption cross sections, poor photostability, and low overall brightness that make single-particle detection and extended biological studies challenging.^13−15^

These limitations can be addressed by incorporating fluorescent inorganic nanoparticles, such as nanocrystal quantum dots (QDs), into VLNPs.^16−18^ Extensive work has been conducted on several QD material compositions, such as the CdSe/CdS core/shell heterostructures used in this work.^19−21^ These advancements in the field have shown that compared to conventional fluorophores, QDs exhibit enhanced photophysical properties that result in improved sensitivity for biosensing and bioimaging applications.^13,22^ This improved sensitivity is complemented by a robust body of work demonstrating excellent stability and specificity of biological probes incorporating QDs.^23−25^ VLNPs incorporating QDs have been previously constructed and have been used to measure viral infectivity, tumor diagnostics, and drug delivery.^17,26,27^ The superior optical properties of QDs in such VLNP applications improved the signal and sensitivity of the assays to probe cellular interactions with the virus-mimicking constructs. However, these constructs either involved direct ligand exchange to introduce a surface protein of interest, or encapsulation into complete envelopes of pseudotyped viruses. In the context of investigating SARS-CoV-2-induced brain inflammation due to physicochemical interactions, the former of these methods may oversimplify the biophysical context of the virus surface, such as the hardness of the surface and the density of proteins. Also, surface capping ligand exchanges can potentially reduce the quality of the QD surface, leading to diminished fluorescence of the VLNP. Conversely, the latter method of construction, which includes all surface proteins and viral genetic content, may make it difficult or even impossible to deconvolute the most essential physicochemical interactions, especially when viral replication in the cell types we used in our models may not be implicated as a key mediator of neurologic disease in COVID-19.^5,6^

Thus, to address how SARS-CoV-2 may initiate brain inflammation, we have designed a SARS-CoV-2 VLNP with a QD core (COVID-QDs). The COVID-QDs were constructed by linking SARS-CoV-2 spike (S) proteins to polymeric phospholipid chains that formed a micellar envelope around the QDs. The design of the COVID-QDs as an enveloped VLNP for SARS-CoV-2 aims to better mimic the surface environment of the SARS-CoV-2 viral envelope to directly interact with the cell; this interaction is lacking in the design of nonenveloped VLNPs, such as a pure QD decorated with S proteins.^17,28,29^ Furthermore, the micellar surface can be tuned to different surface densities of S protein as well as a mixture of other surface proteins by varying the ratio of different variants of the polymeric phospholipid chains. This allows the investigator to create highly adaptable QD VLNPs to mimic key SARS-CoV-2 physicochemical interactions of interest. The COVID-QDs reported here were designed to achieve these effects by accurately replicating the physical dimensions and the average number of surface-bound S proteins reported for SARS-CoV-2 virions.^17,30,31^ Additionally, the inherent photophysical properties of the COVID-QDs allow for fluorescent readout of their localization and interaction with biomolecular targets; properties that are not inherent to most other SARS-CoV-2 virus-like nanoparticle constructs.^32,33^

Our results show that these COVID-QDs structurally and functionally mimicked SARS-CoV-2 virions and elicited an immunologic response in cultured cells without infection. Additionally, we report that COVID-QDs induce loss of endothelial tight junctions and upregulation of inflammatory molecules using an *in vitro* model of the BBB. These changes are reversible with treatment of either soluble human angiotensin-converting enzyme 2 (hACE2), serving as a decoy for the S protein, or URMC-099, an anti-neuroinflammatory, small-molecule treatment.^34−36^ Finally, we observed induction of neuronal synaptodendritic beading, a marker of injury associated with loss of normal neurologic function, in a co-culture model system of the NVU after S or COVID-QD treatment, which also could be reversed with treatment of either hACE2 or URMC-099. Taken together, our data support COVID-QDs are a potent tool to further interrogate the neurological deficits associated with SARS-CoV-2.

## Results and Discussion

### Construction and Characterization of COVID-QDs

**Figure 1:**
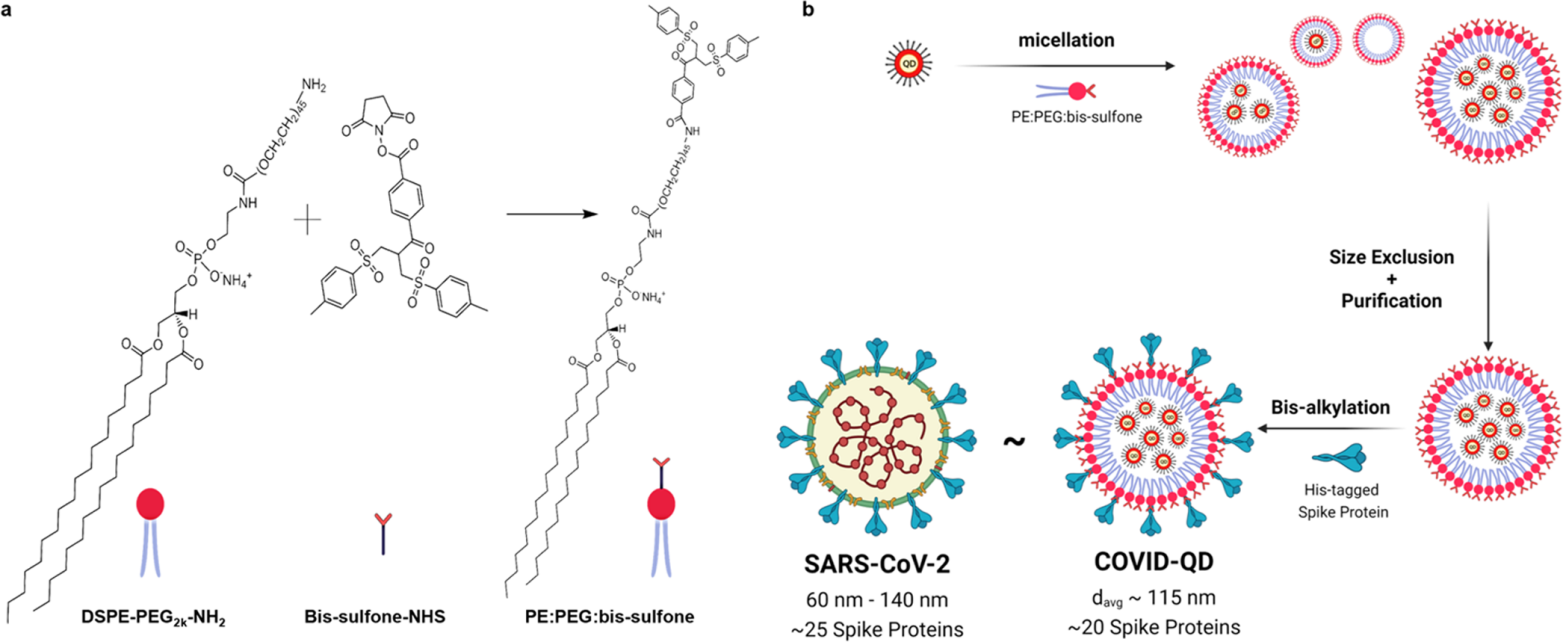
Construction of COVID-QDs. (a) Chemical structure diagram of PE:PEG:bisu-sulfone synthesis. (b) Assembly of QDs into QD-micelles, and conjugation of multiple S proteins onto COVID-QDs. Created with Biorender.com.

**Figure 2:**
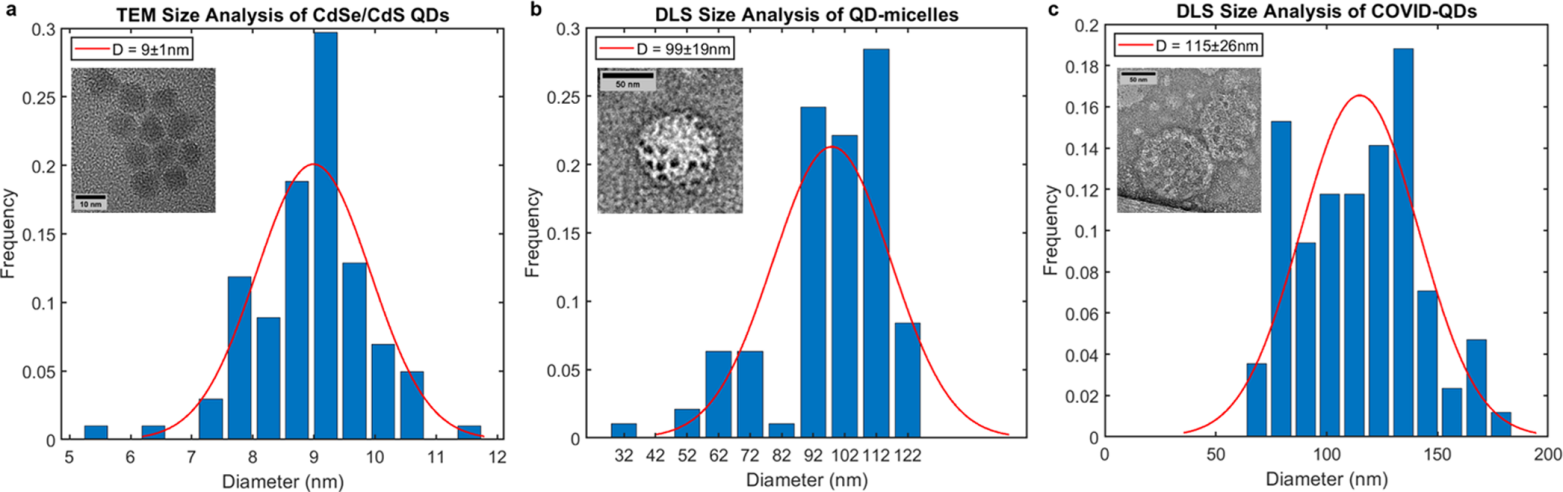
Transmission electron microscopy (TEM) and dynamic light scattering (DLS) analysis of CdSe/CdS QDs, QD-micelles, and COVID-QDs. Gaussian-fitted histograms for size analysis of (a) CdSe/CdS QDs determined by TEM; (b) large QD-micelles eluted fractions used to construct COVID-QDs, determined by DLS; (c) COVID-QDs determined by DLS. The insets in (a–c) are representative TEM images of each (scale bars are 10, 50, and 50 nm, respectively).

The ligand that would form the micellar envelope (PE:PEG:bis-sulfone) was synthesized by appending bis(2-methylphenyl) sulfone (bis-sulfone) functional groups to poly(ethylene glycol) (PEG) and polymerized phosphatidylethanolamine (PE) molecules via an NHS-Ester conjugation reaction (Figures 1a, S1, and S2). This PE:PEG:bis-sulfone construct allows for efficient sequential Michael addition-elimination reaction, or bisalkylation, selective for adjacent imidazole functional groups in polyhistidine chains.^37^ Thus, as diagrammed in Figure 1b, after encapsulation of previously synthesized CdSe/CdS QDs (Figure 2a) into micelles, this bisalkylation mechanism allowed efficient, irreversible conjugation of multiple S proteins to form COVID-QD constructs without risk of perturbing the native tertiary protein structure, and thus providing functional recognition of the S proteins decorating the micellar surface. CdSe/CdS core/shell QDs were the selected material composition for this study given the well-established understanding of the synthesis of these QDs and their resultant photophysical properties,^19−21,38,39^ as well as previous work done by our group and many others related to modifying the surface of CdSe/CdS QDs for biologically relevant applications.^15,21,24^

**Figure 3:**
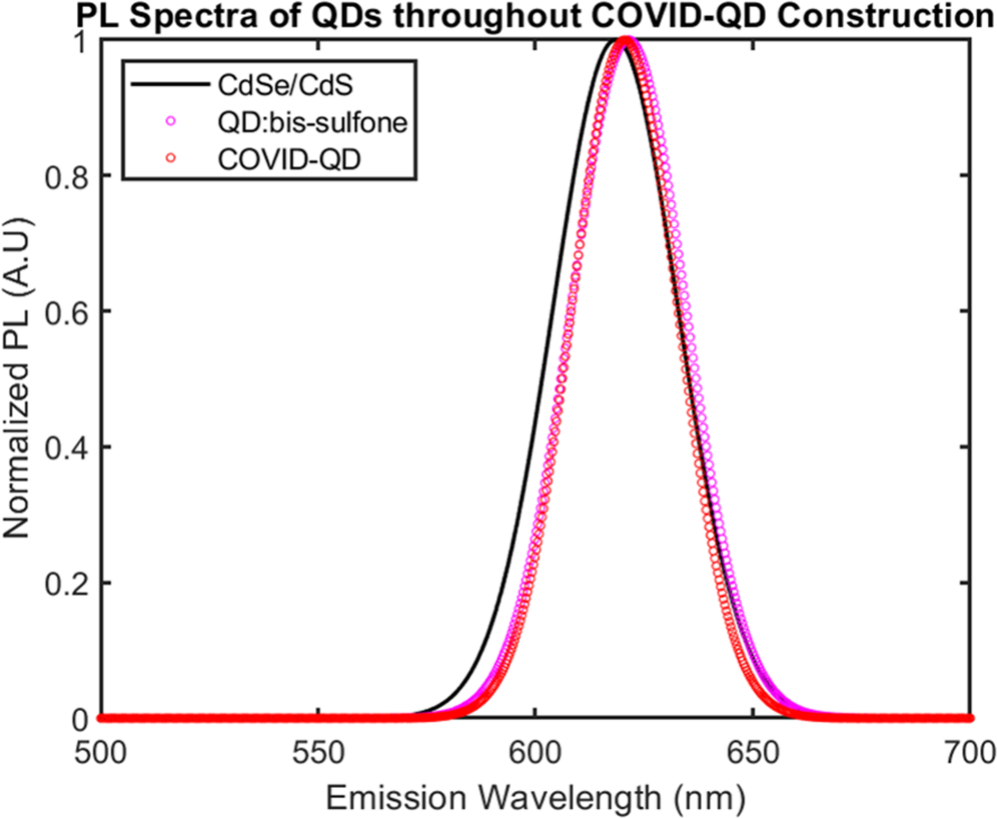
PL spectra of particles after each step. Representative PL spectra from each sample type, depicting minimal shift and change in bandwidth (FWHM ≈ 40 nm) of CdSe/CdS QD emission after micelle encapsulation, size selection, and spike conjugation to construct COVID-QDs.

Absorbance measurements were taken pre- and postencapsulation of CdSe/CdS into PE:PEG:bis-sulfone (QD-micelles) to qualitatively ensure that QD surfaces are not severely modified, as reflected by no apparent changes in the shape and band-edge peak location of the absorbance curve between the CdSe/CdS QD (Figure S3) and that after the CdSe/CdS QD was encapsulated into micelles and COVID-QDs (Figure S4). Additionally, the absorbance features provide insight into the encapsulation efficiency and purity; for example, the QD-micelles exhibit a scattering shoulder at wavelengths higher than the band-edge transition 1S transition peak, attributed to the presence of empty micelles (Figure S4).^40,41^ These were removed from the solution via ultracentrifugation, followed by successive rounds of size-exclusion chromatography to remove smaller QD-micelles (Figure S4). The eluted fractions containing the largest of QD-micelles were determined to have an average hydrodynamic diameter of 99 nm via dynamic light scattering (Figure 2b). Post-conjugation of a 20-times molar excess of S protein, the final COVID-QD constructs had an average hydrodynamic size of 115 nm (Figure 2c); this size falls well within the range of 80–140 nm commonly reported for SARS-CoV-2 virions from cryo-EM and atomic force microscopy (AFM) studies.^30,31^ After termination of the bisalkylation conjugation, we eluted the unbound fraction of S proteins from the COVID-QD solution using a size-exclusion spin column and measured the absorbance at 280 nm (*A*_280_) of the eluted fraction on a NanoDrop Spectrophotometer. The *A*_280_ measurement was corrected for molecular weight and estimated extinction coefficient of S protein to determine a conjugation efficiency of ∼88% for the reaction (Table S1). This represents an average of 18 S proteins/COVID-QD, which is within a previously reported distribution of 24 ± 9 S protein per SARS-CoV-2 virus particle.^42^ The measured parameters of structural mimicry for the COVID-QDs and their unaltered photoluminescent (PL) emission spectra in each step of construction (Figure 3) suggest that this construct may be used to fluorescently probe functional activity similar to native SARS-CoV-2 virus particles.

Lastly, before translating the COVID-QDs into experiments involving cells, we performed transmission electron microscopy (TEM) to examine the stability of the self-assembled micelles around the QDs at the working concentrations of our experiments (≤10 nM QD-micelles). The micelles were constructed with 50,000× molar excess of PE:PEG:bis-sulfone monomers to ensure that after successive rounds of purification (i.e., removal of empty micelles and smaller QD-micelles), there would be sufficient PE:PEG:bis-sulfone monomers in solution to satisfy the critical micelle concentration (CMC) for the phospholipid-PEG backbone (DSPE-PEG_2k_).^43^ Given that the final QD-micelles used to construct the COVID-QDs are larger self-assemblies containing multiple QDs (Figures 2b,c and S4), we expected a large excess of PE:PEG:bis-sulfone monomers to be present in the micellar capsule, thus allowing for stable micelles to be present at ≤10 nM concentration of micelles, which would correspond to PE:PEG:bis-sulfone monomer concentrations close, but still satisfying the lower CMC limit for DSPE-PEG_2k_.^43^ This is reflected by the presence of relatively symmetrically shaped lightly shaded micellar particles on negatively stained TEM grids, with multiple darker, high electron-density QDs encapsulated within each micelle (Figures 2b and S4). Additionally, a body of work by others have characterized the stability of inorganic nanoparticles encapsulated by lipid-PEG micelles to suggest that the intercalation of the lipid moieties of the micelle with the capping ligands on the nanoparticle surface may further stabilize the self-assembled micellar construct to allow for lowered CMC conditions.^44−48^ As a control, a set of QD-micelles were constructed using a far lower molar excess of PE:PEG:bis-sulfone monomers (<5000×) and was subjected to the same rounds of purification to isolate larger QD-micelles. These were not stable in aqueous solution for extended periods of time and would pellet out of solution. While vortexing and sonication could promote temporary resuspension, TEM analysis showed these micellar constructs to be unevenly shaped and highly prone to CdSe/CdS QDs embedded within polymeric aggregates (Figure S5). Thus, only stable suspensions of larger QD-micelles and COVID-QDs were used for the biological experiments covered in this work.

### COVID-QDs Dysregulate bEnd.3 Monolayers

**Figure 4:**
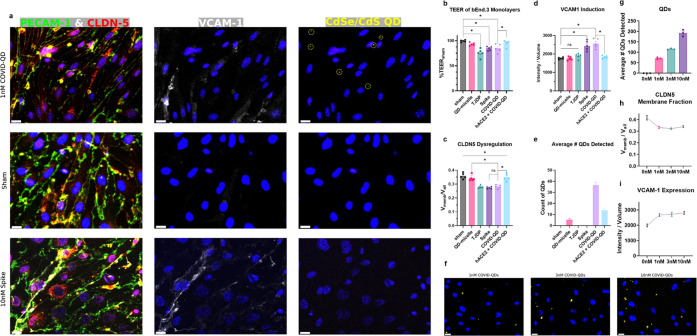
Evaluation of immunomodulation of bEnd.3 monolayers. (a) Effects of S protein and COVID-QDs on localization of CLDN-5 and expression of VCAM-1 compared to sham, media-only treatments. Nuclei are stained by 4′,6-diamidino-2-phenylindole (DAPI) (dark blue) in all micrographs. Intracellular clusters of CLDN-5 that are not membrane-localized are demarcated by arrows. COVID-QD PL emission is demarcated with dashed circles centered around the QD puncta. Scale bar, 15 μm. (b) TEER measurements of monolayer integrity. (c) Changes in membrane-localized fraction of CLDN-5 compared to total CLDN-5. (d) Changes in VCAM-1 expression level based on average intensity level distributed across all VCAM-1+ regions. (e) Average number of QD constructs detected from micrographs associated with each treatment group. (f–i) Dilution series dose response of bEnd.3 monolayers to 1, 3, and 10 nM COVID-QD treatments, represented by micrographs of increased QD-associated puncta (f), with increased average number of QD-associated puncta detected per replicate (g), and plateaued responses in loss of CLDN-5 membrane fraction (h), and increase of VCAM-1 expression (i). Statistical analyses shown are mean ± standard error of measurement (SEM), where significance is *p* < 0.05 based on one-way analysis of variance (ANOVA) + Holm-Sidak post hoc and were computed using *n* = 5 wells over two passages.

Functional mimicry of the COVID-QDs was assessed in an *in vitro* BBB model system formed by bEnd.3 monolayers cultured on transwell inserts. Changes in barrier integrity (Figure 4a–c) were determined by a statistically significant reduction in both transendothelial electrical resistance (TEER) and a complementary semi-quantitative immunocytochemical (ICC) analysis of the membrane-localized fraction of tight junction protein claudin 5 (CLDN-5). TEER measures the net flow of ionic species across the monolayer; this flow is represented as a net resistance that decreases as the barrier is damaged. As shown in Figure 4b, treatment with 1 nM COVID-QDs or 10 nM S protein reduced barrier integrity in the bEnd.3 monolayers that were not observed in sham, media-only treatments. Our results agree with previous reports that have shown that soluble S protein elicited inflammation and increased permeability at the brain endothelium.^8,9,49^

Decreases in barrier resistivity should be accompanied by reorganization of membrane-localized tight junction proteins that line the gaps between adjacent endothelial cells. Thus, membrane-localization of CLDN-5 was assessed by fluorescent readout of spatial colocalization with platelet endothelial cell adhesion molecule 1 (PECAM-1), which is constitutively expressed along the membrane of vascular cells.^50,51^ Loss of BBB integrity can manifest as a redistribution of membrane-localized CLDN-5 to increased intracellular clusters that no longer co-localize with PECAM-1 (Figure 4a,c). We also observed marked increases in the fluorescent readout of vascular cell adhesion molecule 1 (VCAM-1) in the bEnd.3 monolayers (Figure 4a,d), which typically exhibits low basal expression of VCAM-1. Upregulation of VCAM-1 expression in bEnd.3 cells by S protein and COVID-QDs indicates endothelial inflammation but does not necessarily correlate with CLDN-5 expression or distribution.

To assess whether the effects of the COVID-QDs were specific to the S proteins on its surface, we treated bEnd.3 monolayers with either unfunctionalized QD-micelles or a mixture of both 1 nM COVID-QDs and 10 nM of soluble hACE2 (Figure S6). The resultant TEER and ICC results (Figure 4b–d) showed no significant decreases in TEER, nor membrane-localized fraction of CLDN-5 and no significant increase in VCAM-1 expression. These results indicate no significant disruption of the bEnd.3 monolayer due to bare QD-micelles and confirms that the micellar construct itself was not responsible for the COVID-QD effects. Moreover, these effects were attenuated by co-treatment with soluble hACE2, a decoy biomolecular target for S protein; this is indicative of the fact that the COVID-QDs are mediating an effect in an S protein-specific interaction.

These observations were corroborated by the density of CdSe/CdS QD PL (Figure 4e) in the optical images. Specifically, COVID-QDs showed the largest amount of QD PL per optical image, while bare QD-micelles exhibited nearly negligible nonspecific binding to the bEnd.3 monolayers as evidenced by significantly reduced QD PL. Also, co-treatment of COVID-QDs with soluble hACE2 resulted in a marked attenuation of COVID-QD binding and detection (Figure 4e). This lack of nonspecific binding suggests that the COVID-QDs are directly interacting with the bEnd.3 monolayers in an S protein-specific manner to mediate the effects observed in Figure 4. Additionally, we performed a logarithmic dilution series of 1, 3, and 10 nM COVID-QDs, corresponding to 10^0^, 10^0.5^, and 10^1^ nM, to the bEnd.3 monolayers to recapitulate the saturation of cellular response to free S protein previously observed.^9^ Despite the increased number of binding events, indicated by an increased number of QD-associated PL in the image (Figure 4f,g), the dysregulation of the membrane-bound fraction of CLDN-5 and increase of VCAM-1 expression levels are not significantly different (*p* > 0.05, one-way ANOVA + Holm-Sidak post hoc) between the three treatment groups (Figures 4h,i and S7). Thus, we conclude that the mimetic capacity of the COVID-QD constructs match that of functional responses from brain endothelial monolayers in response to similar doses of S protein.

We further confirmed that the effects we observed are unique to S protein by treating the bEnd.3 monolayers with a tight junction-disrupting peptide (TJDP, Figure S8).^52^ As expected, damage to endothelial tight junction integrity by TJDP treatment resulted in a marked decrease in TEER and membrane-localized fraction of CLDN-5 (Figure 4b–d), but, in contrast to S protein or COVID-QD application, did not change VCAM-1 expression (Figure 4d). This suggests that the inflammation of bEnd.3 monolayer due to COVID-QDs are specifically in response to S protein and not simply a byproduct of reduced barrier integrity. As final validation of our hypothesis, we performed a resazurin assay as an index of an intact mitochondrial respiratory chain in viable endothelial cells to confirm that these functional phenotypic changes are not simply a cytotoxic effect from either COVID-QDs or S protein. As shown in Figure S8, we did not observe any significant difference in the viability of bEnd.3 cell treatment groups in response to our TJDP, Spike, or COVID-QDs, nor from any of the other treatment groups involved later in this study compared to our sham negative control group.

### BBB Damage Results in Neuroinflammation

**Figure 5:**
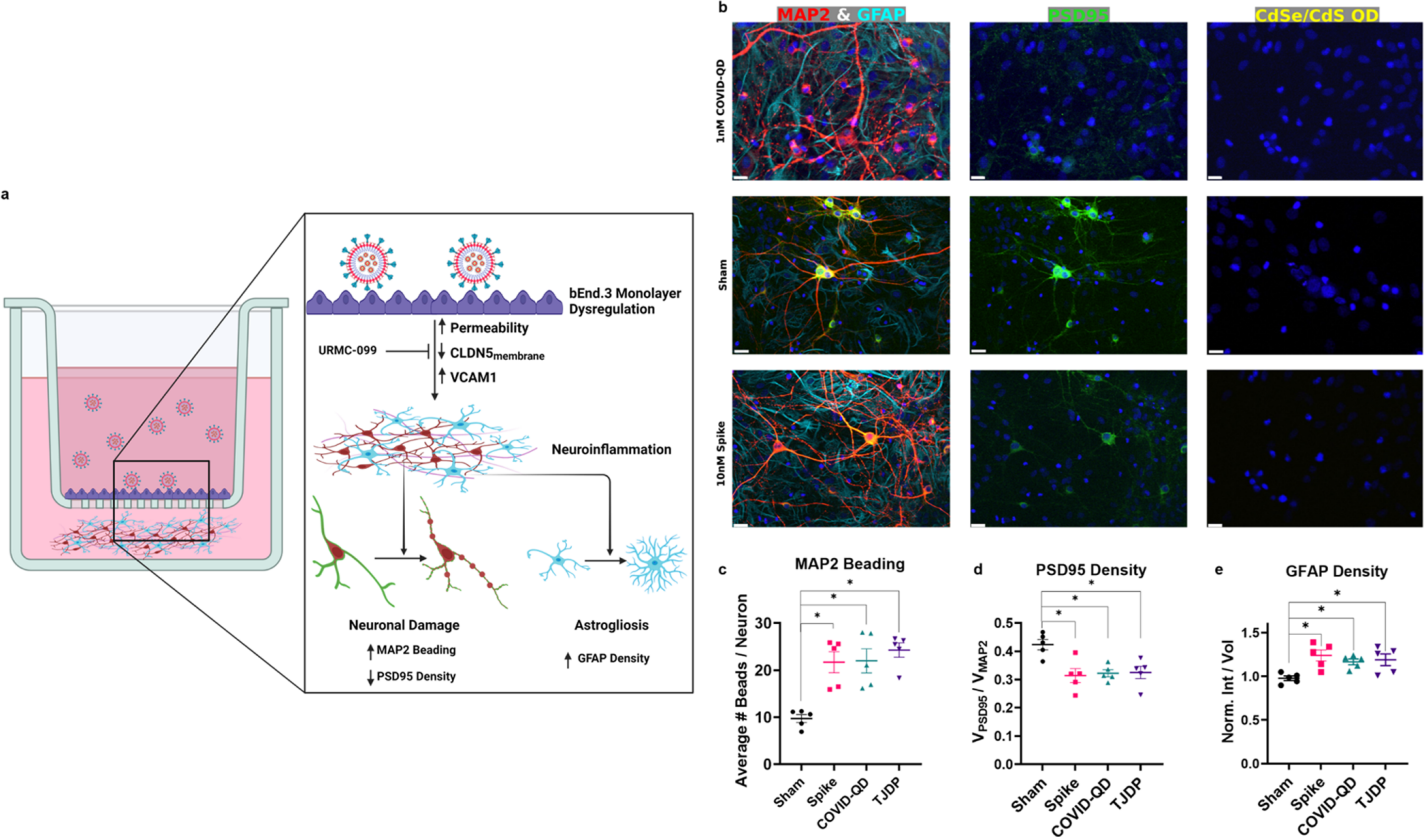
Changes in neuroinflammatory state of neuroglial culture in response to different treatment groups applied to bEnd.3 monolayers. (a) Cartoon diagraming transwell co-culture model of static NVU and expected neuroinflammatory hallmarks. Created with Biorender. (b) Immunofluorescent labeling of neuronal markers (red MAP2 and green PSD-95), astroglia marker (cyan GFAP), and QD fluorescence (yellow puncta). DAPI-stained nuclei shown in all panels. Scale bar = 15 μm. (c) Quantification of the average number of dendritic beads formed per neuron. (d) Quantification of postsynaptic densities based on spatial distribution in all neuron+ regions. (e) Quantification of astrogliosis based on the expression of GFAP determined by average intensity across all GFAP+ regions. Plots shown are mean ± SEM, where significance is defined as *p* < 0.05 based on one-way ANOVA + Holm-Sidak post hoc and *n* = 5 cultures pooled from two trials.

We constructed a static model of the NVU assembling bEnd.3 monolayers cultured on transwell cell culture inserts above cultured primary rat hippocampal neurons and astrocytes cultured on coverslips in multiwell plates (Figure 5a). The bEnd.3 monolayers act as a model BBB and segregate the basolateral (upper chamber) media from that of the apical (lower chamber) media that is conditioned with soluble factors released by the neuroglial culture. Thus, by applying treatments only to the basolateral media, we were able to isolate how changes in monolayer integrity and health resulted in subsequent immunomodulation of neuroglial health. Specifically, we expected that the leakage across the monolayers and potential activation of pro-inflammatory signaling events would induce neuroinflammation in the mixed neuroglial cultures.^53,54^ To evaluate cellular damage from neuroinflammation, we immunolabeled neurons for microtubule-associated protein 2 (MAP-2) and postsynaptic density 95 (PSD-95) and astrocytes with glial fibrillary acidic protein (GFAP). The first two markers define subcellular neuronal elements necessary for normal signaling between neurons at synapses, and GFAP expression can define abnormal responses to inflammation. As shown in Figure 5a,b, these markers demonstrate hallmarks of neuronal injury that can be associated with excitotoxicity and neuroinflammation: dendritic beading, synaptic pruning, and astrogliosis, respectively.^55−57^

In agreement with our hypothesis, the dual effect of increased barrier leakage and inflammatory state of the bEnd.3 monolayers in response to 10 nM S protein or 1 nM COVID-QDs resulted in significant dendritic beading in MAP-2 labeled neurons (Figure 5c), a common hallmark of neuronal injury associated with reduced efficiency of signal propagation along processes. This is indicated by the presentation of focal swellings, or punctate discontinuities, in the dendrites stained by MAP2 in Figure 5b, and was accompanied by reorganization of dendritic spines, as represented by a significant decrease in the spatial density of PSD-95 labeling (Figure 5d). The loss of postsynaptic densities is indicative of synaptodendritic injury, and reflects decreased sites for signal transmission between neurons.^56,58^ The combination of both these hallmarks associated with reduced signal propagation and transmission may be linked to the observed cognitive decline during COVID-19 and long COVID patients.

Additionally, we found evidence for an increase in the density of astrocytes (i.e., reactive astrogliosis) indicated by an increase in average GFAP intensity and density (Figure 5e); this is visually reflected by the increased cyan features in Figure 5b. Astrogliosis could occur because astrocytes are potential targets for SARS-CoV-2 in the CNS through binding of membrane-bound receptors (such as neuropilin-1).^59,60^ Alternatively, activation of the bEnd.3 monolayers may result in the observed reactive astrogliosis.^61,62^ This activation of astroglia is consistent with neuroinflammation and explains the observed synaptic decrease of PSD-95 (Figure 5d), where activated glial cells in a neuroinflammatory state may engulf and prune synapses.^57,63^ It is notable that fluorescent micrographs for COVID-QD PL yielded no substantial evidence for transmigration of the nanoparticles across the dysregulated bEnd.3 monolayers and, thereby, no subsequent binding to astroglia (Figure 5b). While this does not rule out the potential for soluble S protein to be transported via transcytosis across the membrane, the lack of COVID-QD PL would thus suggest that the presence of SARS-CoV-2 virions in the CNS is not necessary to drive neuroinflammation.

### URMC-099 Protects against Dysregulation of NVU

**Figure 6:**
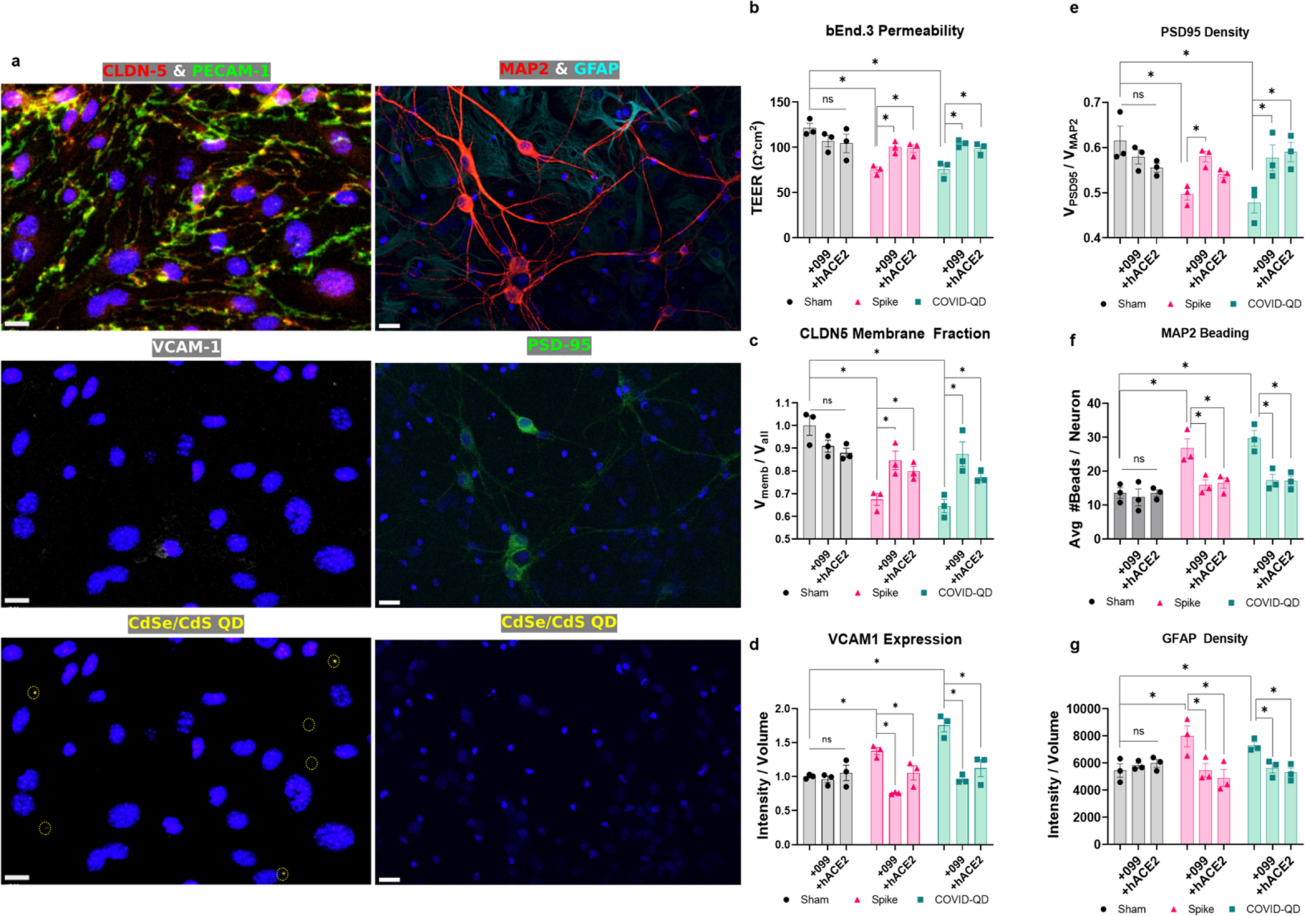
Rescue of disrupted endothelial monolayer and subsequent neuroinflammation. (a) Representative fluorescent micrographs of bEnd.3 cells and neuroglia in co-culture in response to URMC-099 pretreatment to rescue COVID-QD-induced damage and inflammation. Nuclei are stained with DAPI in dark blue (scale bar = 15 μm). (b–d) Readout of rescue of bEnd.3 monolayer health by TEER (b), CLDN-5 membrane-localized fraction (c), and VCAM-1 expression levels (d). (e–g) Readout of corresponding mitigation of neuroinflammation by PSD-95 density (e), MAP-2 beading (f), and GFAP expression levels and density (g). Significance defined as *p* < 0.05 tested by two-way ANOVA with Holm-Sidak post hoc correction and *n* = 3.

Our observations thus far implicate dysregulation of bEnd.3 monolayer integrity and inflammation as a driver of downstream neuroinflammation. Thus, we introduced either a pretreatment of 200 nM URMC-099 or co-treatment with 10 nM soluble hACE2 to our 10 nM S protein or 1 nM COVID-QD treatments in the co-culture model NVU system (Figures 6 and S10–S13). URMC-099 is a small-molecule therapeutic that inhibits mixed lineage kinase type 3 and leucine-rich repeat kinase type 2, responsible for a broad spectrum of inflammatory responses in the NVU and has been previously validated in various acute and chronic neurologic disease models.^34,35^ Thus, pretreatment with URMC-099 is expected to attenuate the deleterious effects of S protein and COVID-QDs on the bEnd.3 monolayer health, and subsequently reduce the formation of neuroinflammatory hallmarks in the neuroglial cultures (Figures 5a and 6a). In line with these expectations, pretreatment with 200 nM URMC-099 significantly reduced (*p* < 0.05, two-way ANOVA + Holm-Sidak post hoc), inflammatory activation and barrier remodeling in the bEnd.3 monolayers (Figure 6b–d). Specifically, immunofluorescent imaging of the monolayers exhibited no observable inflammatory induction of VCAM-1 and increased membrane-localization of CLDN-5, matching what is seen in healthy, untreated monolayers in Figure 6a. This is complemented by improved TEER measurements (Figure 6b), indicative of healthier barrier function. Correspondingly, immunofluorescent analysis of the neuroglia populations in co-culture with these endothelial monolayers presented less neuroinflammatory hallmarks—lowered amounts of dendritic beading, reduced spreading of astrocytic processes (i.e., astrogliosis), and increased numbers of synaptic densities (Figure 6a,e–g). Likewise, as soluble hACE2 acts as a decoy to reduce COVID-QD interaction with the endothelial monolayer, pretreatment with soluble hACE2 should also reduce downstream neuroinflammatory events, which we demonstrate via pretreatment with 10 nM of soluble hACE2 (Figures 6b–g, S11, and S12).

The rescue of barrier health and mitigation of neuroinflammation with soluble hACE2 validates that (i) our COVID-QD constructs are functionally recognized as a SARS-CoV-2-like nanoparticle and that (ii) the observed neuroinflammatory events in response to COVID-QDs are likely in response to inflammation of the endothelium and not via direct interactions with S protein. Additionally, while soluble hACE2 has been theorized as a potential treatment to mitigate general SARS-CoV-2 pathogenesis, its use is complicated by a short half-life in peripheral circulation.^64,65^ In that regard, our observed rescue of NVU health with URMC-099 pretreatment suggests a potential adjunctive therapy that could be applied to COVID-19-associated neurologic disease.

## Conclusions

We constructed a high-fidelity structural and functional mimic for native SARS-CoV-2 virus particles using a CdSe/CdS QD core; this mimic provided a fluorescent readout relevant to elucidating the potential role of virus particle transmigration into the CNS to induce neuroinflammation. Fluorescence from COVID-QDs combined with an immunocytochemical readout and functional TEER assays of a model NVU co-culture demonstrated that endothelial inflammation and leakage were sufficient to induce the formation of neuroinflammatory hallmarks without the transmigration of COVID-QDs across bEnd.3 monolayers. Various studies have implicated the induction of neuroinflammation as a likely mediator of altered neurologic function associated with COVID-19 neuropathophysiology.^5,66^ In line with these hypotheses, our data provides support for a proposed model for neurological manifestations of COVID-19 *via* a dysregulated BBB through direct physiochemical interactions of SARS-CoV-2 VLNPs with the endothelium.^7,9^ In addition, we showed that with either soluble hACE2 as a decoy biomolecular target or with URMC-099 as an adjunctive anti-inflammatory therapeutic, endothelial dysregulation and subsequent neuroinflammation are significantly attenuated. This is represented by the improvement of barrier function in the endothelial monolayers, represented by an increase in TEER measurements (Figure 6b), indicating less aberrant flow across, complemented by increased observations of membrane-localized CLDN-5 (Figure 6c), a tight junctional protein associated with regulating barrier integrity. The improved barrier health is complemented by reduced inflammation in the endothelial monolayers, detected via reduced induction of VCAM-1 expression, a marker for inflammatory activation (Figure 6d). These improvements in endothelial barrier health due to soluble hACE2 and URMC-099 subsequently improves the health of the neuroglial cultures, reflected by restored postsynaptic densities (Figure 6e), reduced observations of dendritic beading (Figure 6f), and reduced astrogliosis (Figure 6g).

In the broader context of further interrogating the neurological manifestations of COVID-19 infection and post-acute infection, the COVID-QDs constructed in this study may be applied to interrogate the interplay between virus particles and neuro-immune signaling at the NVU. This highlights a limitation to the biomedical conclusions that may be inferred from the results of our work. While our co-culture model of the NVU contains key cellular regulators, it does not fully recapitulate the complete cellular architecture of a true, dynamic NVU. As such, a larger, more complex interaction network between such cell types and SARS-CoV-2 virus particles are likely potent contributors to COVID-19 neuropathophysiology and are of interest in future studies with respect to long COVID and other neurologic sequelae. In line with such motivations, one outstanding question of *in vitro* models is always about how translatable the observations may be in a living organism. Thus, ongoing work is beginning to apply our biomimetic COVID-QDs to mouse models of moderate to severe COVID-19.

## Materials and Methods

### Synthesis of CdSe Cores

#### Reagents

All reagents were used as purchased from the manufacturer without further purification. The following were purchased from Sigma-Aldrich: trioctylephosphine (TOP; cat: 718165), selenium pellets (Se; cat: 209643), diphenylphosphine (DPP; cat: 252964), cadmium oxide (CdO; cat: 202894), trioctylphosphine oxide (TOPO; cat: 223301), 1-hexadecylamine (HDA; cat: 445312), tetradecylphosphonic acid (TDPA; cat: 736414).

#### 1 M TOP:Se

Prior to synthesis, 1 M TOP:Se was synthesized following a protocol as previously reported.^15^ In a glovebox, 680 mg of Se pellets and 8.5 mL of TOP were combined in a scintillation vial and mixed at 60 °C overnight to result in a clear solution. A small amount of secondary phosphines, 90 μL of DPP, was added to help promote efficient QD nucleation.^67^

#### Protocol

The CdSe cores for the QDs were synthesized using a procedure adapted from those previously reported.^15,68^ We added 820 mg of CdO, 16.2 g of TOPO, 37 g of HDA, and 3.2 g of TDPA to a 250 mL 3-neck flask and heated the flask to 90 °C under N_2_. The flask was then degassed by 3 cycles of evacuation (<100 mT) and refiling with N_2_ before leaving the mixture under N_2_ and heating it to 320 °C with rapid stirring. Complexation of Cd-TDPA was visually determined by the transition from an opaque to translucent solution, upon which the flask was cooled to 260 °C. While maintaining rapid stirring, 8.0 mL of 1 M TOP:Se was rapidly injected and the cores were grown for 2–3 h until the desired size was achieved. This was determined by taking PL measurements of aliquots every 10 min after the 2 h timepoint. The reaction was then quenched by removing the flask from heat and applying forced air to bring the solution to 200 °C before submerging the flask into a water bath to rapidly cool the solution to 100 °C. The solution was then injected with 40 mL of ButOH and allowed to cool for ∼1 h before performing several rounds of washing followed by two cycles of size-selective precipitation. The final pellet was resuspended in hexane and filtered through a 0.45 μm syringe filter to produce the CdSe stock used for shelling.

### Shelling of CdSe/CdS QDs

#### Reagents

All reagents were used as purchased from the manufacturer without further purification. The following were purchased from Sigma-Aldrich: oleic acid (OAc; cat: 364525), octadecylamine (ODA; cat: 74750), oleylamine (OAm; cat: O7805), octadecene (ODE; cat: O806), octanethiol (OT; cat: 471836).

#### Cd-Oleate Preparation

Prior to synthesis, Cd-oleate was prepared similar to previously reported.^15,67^ To a flask were added 250 mg of CdO, 2.6 mL of OAc, and 20 mL of ODA. The contents were degassed at room temperature, followed by heating to 270 °C for 90 min under N_2_. The flask was then cooled to 150 °C and injected with 1.3 mL of OAm. The Cd-oleate product was stored in a glovebox until needed.

#### Protocol

The CdS shelling procedure is a modified protocol from that previously reported,^15,68^ where 100 nmol of the CdSe stock solution, 3 mL of OAm, and 3 mL of ODE were added to a 100 mL 3-neck flask and degassed at room temperature for ∼1 h under constant stirring at 800 rpm. The CdSe mixture was then kept under vacuum and stirred while heated to 115 °C for 20 min. The flask was then refilled with N_2_ and heated to 350 °C at a ramp rate of 16 °C/min. During this time the Cd and S precursors were individually loaded into two separate syringes, where 0.150 mmol of Cd-oleate (∼2.2 mL) was diluted to 3.5 mL with ODE in one syringe and 0.180 mmol of OT (∼0.04 mL) was diluted to 3.5 mL with ODE in the other. The syringes were affixed to a dual syringe pump and injected at a rate of 1.5 mL/h once the solution reached 200 °C. The reaction was held at 350 °C for shell growth before cooling to 200 °C and followed with dropwise addition of 1 mL of OAc. The solution was left to anneal for 1 h, cooled to 75 °C, then transferred to falcon tubes for three rounds of washing. The final pellet was resuspended in either hexane or toluene and stored in a glovebox.

### Synthesis of DSPE-PEG_2k_-bis(2-methylphenyl)sulfone (PE:PEG:Bis-Sulfone)

#### Reagents

All reagents were used as provided by the manufacturers without further purification. The polymeric phospholipid was purchased from Nanocs, Inc. (DSPE-PEG_2k_-NH_2_; cat: PG2-AMDS-2k). The bis(2-methylphenyl)sulfone-NHS-ester was purchased from BroadPharm (Bis-sulfone NHS Ester; cat: BP-23344). Triethylamine (TEA; cat: 15791) was purchased from Acros Organics.

#### Protocol

The synthesis of PE:PEG:bis-sulfone was carried out via a modified NHS-ester crosslinker conjugation in dichloromethane, adapted from Zhang et al.^69^ In a 1:1 mole ratio, DSPE-PEG_2k_-NH_2_ and bis-sulfone NHS ester were added to a 25 mL 2-neck flask and degassed for 1 h at room temperature. During this time a 10 mole excess of TEA was diluted in dichloromethane until an approximate pH of 8.5 was achieved using a pH strip pre-wetted with nanopure water. The flask was then refilled with N_2_ and the TEA solution was slowly injected. The reaction was left under N_2_ and gentle stirring (∼300 rpm) for 24–48 h until the reaction was determined to be complete. The reaction progress was monitored by thin-layer chromatography by using 7.5% (v/v) methanol:chloroform as the eluting solvent and short-wave UV irradiation to visualize the migration of the spotted aliquots and reagents. Matrix-assisted laser desorption/ionization-time of flight (MALDI-ToF) (Figure S1) and NMR (Figure S2) were used to confirm that the desired product was formed.

#### MALDI-ToF

1 μL of the product was spotted for MALDI with 1 μL of a matrix composed of α-cyano-4-hydroxycinnamic acid, 0.5% trifluoroacetic acid, and 0.1% NaCl dissolved in EtOH and measured at 60–80% power (Shimadzu Axima Performance).

#### NMR

For NMR, the product was dried using a rotary evaporator and redissolved in CDCl_3_ with a small aliquot of toluene added as an internal reference standard to determine a relative concentration using ^1^H NMR peak integration. All ^1^H and ^13^C spectra were acquired using an Avance 500 (Bruker) at 298 K, and the resultant peaks were manually integrated.

### Construction of COVID-QDs

#### Encapsulation of QDs in PE:PEG:Bis-Sulfone Micelles

An aliquot of CdSe/CdS core/shell QDs was dried under a rotary evaporator and resuspended in CHCl_3_. The QDs and PE:PEG:bis-sulfone reagents were separately aliquoted at a 1:50,000 mole ratio and briefly sonicated for 2 min (power level 6, VWR; model 250D) to disperse any small QD clusters or preformed micellar structures. The two reagents were then combined in a vial and diluted to 10× with CHCl_3_ before briefly vortex mixing for 1 min followed by sonication for 3 min. The solution was then dried under a rotary evaporator. The dried gel-like film was then briefly annealed at 80 °C in an oil bath for 10 min and then resuspended in ddH_2_O and stirred at 300 rpm for 1–3 h at RT. Following this, the QD-micelle solution was then passed through a size-exclusion spin column (Cytiva; cat: 27513001) to remove any unencapsulated QDs, free PE:PEG:bis-sulfone ligands, or overtly large micelles. This should result in a clear solution that is then passed through a 100k MWCO Amicon Ultra Centrifugal filter (Millipore Sigma; cat: UFC810024) was used to concentrate down the QD-micelle solution and remove any QD-micelles that would be too small to replicate a natural SARS-CoV-2 virion. This product was then diluted 4× with ddH_2_O and then centrifuged at 16,000*g* for 45 min to isolate to distinct size groups of QD-micelles. The pellet was resuspended with ddH_2_O. The eluent from the 100k MWCO filter, the supernatant, and the resuspended pellet were spotted onto a 384-well microplate and characterized with dynamic light scattering (Wyatt DynaPro Plate Reader II) and photoluminescent spectra. Photoluminescent spectra of QD, QD-micelle, and COVID-QD were imported onto MATLAB and fitted with Gaussian approximations, normalized, and plotted as shown in Figure 2.

#### Dynamic Light Scattering (DLS)

The DynaPro system was controlled using the Dynamics software (Wyatt; v7.10) to acquire and preliminarily process the gathered spectra. Each sample was spotted in two separate wells with ten 3 s acquisitions being acquired over each well per run and each run being repeated three times. The resultant acquisitions were processed using the “Legacy” fitting algorithm on Dynamics and then filtered for further analysis based on manual inspection of the resultant correlogram for each individual acquisition. The remaining acquisitions were then imported onto MATLAB to be plotted into a frequency-normalized histogram with a Gaussian-fitted distribution overlayed as shown in Figure 2.

#### Conjugation of Spike Protein to QD-Micelles

Using the results of the dynamic light scattering analysis, the solution containing the optimal size distribution best matching native SARS-CoV-2 virions was selected. To get a rough approximation of concentration, the dilution-corrected intensity of the PL was compared to that of the QDs before micelle encapsulation. Before introducing the Spike protein, the bis-sulfone groups on the QD-micelle surface were activated by an elimination reaction to produce the mono-sulfone form of the ligand that then undergoes the bisalkylation conjugation with the poly-His tag on the Spike protein (Invitrogen; cat: RP-87668).^37^ This was done by concentrating down the QD-micelles using a 30k MWC Amicon Ultra Centrifugal filter (Millipore Sigma; cat: UFC203024) and diluting 4× in a 50 mM sodium phosphate buffer pH 7.4 + 100 mM NaCl. The solution was then incubated at 37 °C for 4–8 h and then a 20x molar excess of Spike protein dissolved in 50 mM sodium acetate buffer pH 5.6 + 35 μM hydroquinone was added. This solution was mixed on a rocker for 18 h at RT. The reaction was then quenched with 1 mM sodium borohydride at 4 °C for 90 min. When the solution was then filtered with a 150k MWCO centrifugal protein concentrator (Thermo Scientific; cat: 89920) to remove any unconjugated Spike protein. The concentrated COVID-QD product was then stored at 4 °C and used within 48 h for biological experiments. The eluent containing the free Spike protein was then concentrated down with a 30k MWCO Amicon Ultra Centrifugal filter (Millipore Sigma; cat: UFC203024) and then spotted onto a NanoDrop Spectrophotometer for an absorbance@280 nm (*A*_280_) measurement corrected for molecular weight and estimated extinction coefficient to determine concentration. This is represented by the following equations, where *A*_280_ is the absorbance value measured at 280 nm, ε (M^–1^ cm^–1^) is the extinction coefficient for the His-tagged Spike protein, *l* (cm) is the path length of the NanoDrop system, and *c* (M) is the associated concentration. The predicted extinction coefficient is calculated based on the number of tryptophan (W), tyrosine (Y), and cysteine (C) residues in the protein sequence. The path length for the NanoDrop system is approximately 0.1 mm.
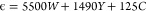

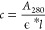

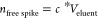

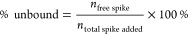


#### Estimation of COVID-QD Concentration

Absorbance measurements of the QD-micelle solutions and final COVID-QD solutions after each purification step to keep track of loss of particles. Spectra were taken on a PerkinElmer Lambda 950 UV–vis–NIR spectrophotometer. The band-edge 1S transition peak was extracted from each spectrum and fitted to empirically determined equations to determine an extinction coefficient for CdSe nanocrystals, which was then used to approximate a concentration of CdSe/CdS QDs in the micellar solutions. The QD concentrations were then scaled down by a factor of 8, reflecting the average number of CdSe/CdS QDs observed to be encapsulated in large QD-micelles used to construct the final COVID-QDs.

### Transmission Electron Microscopy (TEM) Analysis of Particles

#### Sample Preparation

10 μL of nanoparticle solutions was drop-cast onto ultrathin lacey carbon-supported copper grids with a mesh size of 400 (Ted Pella; cat: 01824). For CdSe/CdS QDs in organic solvents, the solution was diluted to an optical density <0.2 before spotting onto the grid, which was covered from light and allowed to dry for at least 90 min before imaging. For QD-micelle and COVID-QD solutions in aqueous solution at various concentrations, after 10 μL of the solution was drop-cast on the grid, the droplet was blotted off using a Whatman #1 filter paper after 3–5 min. This was followed by 10 μL of 1% uranyl acetate that was blotted off after 1 min with the same filter paper. This was then covered to protect it from ambient light and allowed to dry overnight before imaging.

#### Sample Imaging

TEM images were collected on an FEI TECNAI F-20 field electron microscope using an accelerating voltage of 200 kV. Energy-dispersive X-ray (EDX) spectra were collected using an EDAX-Octane T Si detector (SDD) spectrometer integrated with the TECNAI F-20 S/TEM system.

#### Image Processing

Images were loaded for analysis and labeling on ImageJ-2 (National Institute of Health). Extracted size distributions were loaded onto MATLAB for statistical analysis, Gaussian fitting, and plotting.

### Cell Culture

#### bEnd.3 Cell Line

The immortalized murine brain endothelial cell line (bEnd.3) was purchased from ATCC (cat: CRL-2299) and maintained according to manufacturer protocols. Specifically, the bEnd.3 cells between passage numbers 24–30 were maintained in a flask containing Dulbeco’s Modified Eagle Medium (DMEM, ThermoFisher; cat: 10567022) + 10% fetal bovine serum (FBS, Atlas Biologicals; cat: F-0500-D) and passaged every 3 days with 0.25% trypsin-EDTA (Thermo Fisher; cat: 25200056). For resazurin viability assays, the bEnd.3 cells were subcultured onto a 96-well plate (Corning; cat: 353377) at a density of 35k cells/well. For use in biological experiments, the bEnd.3 cells were subcultured onto 0.4 μm pore diameter PET transwell inserts (Greiner Bio-One; cat: 662640) at a seeding density of ∼85k cells/well. The formation of monolayers on these inserts was assessed daily by examining the confluency of the cells under a light microscope and taking complementary transendothelial electrical resistance (TEER) measurements (World Precision Instruments; EVOM2) after confluency appeared to be at least 80%. Monolayers of greater than 90% confluency and stagnating increases in TEER were then used in biological experiments.

#### Rat Hippocampal Neuroglial 1° Culture

Sprague Dawley (SD) rats at embryonic day 18 (E18) were used to prepare the primary neuroglial cultures for the *in vitro* model neurovascular unit setup in these experiments. The care and use of these animals were in accordance with the Guide for the Care and Use of Laboratory Animals, with protocols approved by the University Committee on Animal Resources at the University of Rochester. Hippocampi were dissected from the E18 SD rats and dissociated in 0.25% trypsin, followed by seeding of the cells at a density of 45k cells/well onto poly-d-lysine coated coverslips (Neuvitro; cat: GG-12–15H) in a 24-well plate. The cells were maintained in neurobasal media (Thermo Fisher; cat: 21103049) supplemented with B27 with antioxidants, 1% GlutaMAX (ThermoFisher; cat: 35050061), 25 μM glutamic acid, and 5% FBS. Every 3–4 days, half of the conditioned media was aspirated off and replenished with neurobasal media supplemented with B27 without antioxidants and 1% GlutaMAX. The neuroglial cultures were used in biological experiments between 18 and 21 days in vitro.

#### Assembly of Model NVU Co-Culture System

Fully formed bEnd.3 monolayers on transwell inserts were introduced to wells containing neuroglial cultures on coverslips 24 h before use in biological experiments.

### Cell Viability Assay

Changes in cell viability in response to the various treatments used in this study were carried out using an alamarBlue (Thermo Scientific; cat #88952) resazurin metabolism fluorescent assay. The bEnd.3 cells were plated at a cell density of ∼30k cells/well in a 96-well plate (Sigma; cat #CLS3904) in DMEM + 10% FBS 48 h before treatment. After the cells reached >90% confluency, as assessed by light microscopy, the cells were primed for treatment in a reduced serum condition (DMEM + 1% FBS) for 3 h, followed by an overnight (∼18 h) incubation with the selected treatments shown in Figure S9. Specifically, the reagents—a sham no treatment group, 200 nM URMC-099, 10 nM soluble hACE2 (Sino Biological; cat: 10108-H05H), 10 nM Spike protein (Invitrogen; cat: RP-87668), 10 μM TJDP, 1 nM QD-micelles, 1 nM COVID-QDs—were prepared in DMEM + 1% FBS and reflected the unique reagents that the bEnd.3 cultures would be exposed to. At the 15 h timepoint, the alamarBlue reagent was added to each well at a final concentration of 10% (v/v). At the 18 h timepoint, a spectrophotometer plate reader (λ_ex_ = 550 nm, λ_em_ = 590 nm) was used to assess the degree of resazurin metabolism in each treatment group. Blank wells containing only 10% (v/v) alamarBlue in the reduced serum media were used to correct for baseline fluorescence. Viability measurements were conducted over two passages for a total of 7 replicates, with the measured fluorescence normalized to a sham, with no treatment group from each passage to correct for passage-to-passage variability.

### Treatment of Cell Cultures

#### Dysregulation of bEnd.3 Monolayers in Single Culture and NVU Co-Culture

Prior to treatment, the cells were incubated in a reduced serum environment (DMEM + 1% FBS) for 3 h. Following this, the abluminal domain of the bEnd.3 monolayers were exposed to a sham media-only group, 10 nM Spike protein (Invitrogen; cat: RP-87668), 1 nM COVID-QDs, 10 μM TJDP, or 1 nM QD-micelles in DMEM + 1% FBS. The cultures were incubated in these treatments for 18 h. Each independent treatment group was repeated for a total of 5 replicates over two separate passages of bEnd.3 monolayers as well as NVU co-cultures (two passages of bEnd.3 monolayers and 1° neuroglia from separate rats).

#### Small-Molecule Rescue of bEnd.3 Monolayer Health

For soluble hACE2 rescue, an equimolar concentration of soluble hACE2 (Sino Biological; cat: 10108-H05H) was co-incubated with 10 nM Spike protein in DMEM + 1% FBS for 30 min prior to treatment. A 10× molar excess of soluble hACE2 was used for co-incubation with 1 nM COVID-QD treatments to compensate for the multiple Spike proteins conjugated to each construct. As a control, a 10 nM soluble hACE2 treatment group was also used to ensure no basal stimulation or artifact may arise from the presence of exogenous hACE2. For URMC-099 rescue, a 200 nM solution was prepared from a 100 μM stock solution diluted in DMEM + 1% FBS. Prior to treatment with 10 nM Spike protein or 1 nM COVID-QD, the bEnd.3 monolayers were pre-treated with the URMC-099 solution for 1 h. URMC-099 was then aspirated off and replaced with treatment of 200 nM URMC-099 + either 10 nM Spike protein or 1 nM COVID-QDs in DMEM + 1% FBS. A control treatment of just 200 nM URMC-099 was also used to ensure no basal activity due to URMC-099. Rescue experiments involving either small-molecule treatment were performed over 3 independent NVU co-cultures for each treatment. Prior to all treatments, the bEnd.3 monolayers were incubated in a reduced serum condition (DMEM + 1% FBS) for 3 h.

### Transendothelial Electrical Resistance (TEER)

An epithelial volt-ohm meter (World Precision Instruments; EVOM2) was used to take ensemble measurements of conductivity across endothelial cell contacts of bEnd.3 monolayers cultured on transwell inserts. TEER measurements were taken daily after bEnd.3 cultures appeared to have >80% confluency under a light microscope. A similar media composition of above and below the transwell membrane was used when taking TEER measurements and the media were allowed to equilibrate to RT for 30 min prior to measurements to reduce measurement artifacts due to temperature fluctuations. For bEnd.3 transwell cultures in co-culture with neuroglia, the inserts were measured in reduced serum media (DMEM + 1% FBS) prior to the introduction of the transwell inserts into the co-culture. At the end of the treatment period, the transwell inserts were moved to a fresh 24-well culture plate containing DMEM + 1% FBS prior to taking a final TEER measurement. The reported TEER values are the difference between the treatment groups with the sham negative control group multiplied by the area of the transwell membrane.

### Immunocytochemical Analysis

#### Preparation of Immunolabeled Samples

NVU co-cultures were first separated by removing the transwell inserts and placing them into a fresh 24-well plate with DMEM + 1% FBS. Both coverslips containing the primary neuroglia and the transwell inserts with the bEnd.3 monolayers were briefly washed with 1× Dulbecco’s phosphate-buffered saline (DPBS, Thermo Fisher; cat: 14190144), followed by fixation with 4% paraformaldehyde (PFA) in 1× DPBS for 15 min. This was followed by 5 min with 100 mM glycine in 1× DPBS and a 5 min wash with 1× DPBS. The cells were then permeabilized with 0.25% Triton-X (Millipore Sigma; cat: T9284) in 1× DPBS for 15 min, followed by two 5 min washes with 1× DPBS. A 1 h blocking step with 10% bovine serum albumin (BSA, Millipore Sigma; cat: A1470) in 1× DPBS was used after permeabilization and followed by treatment with the relevant primary antibodies (Table S2) in 3% BSA in 1× DPBS overnight on a rocker at 4 °C. The next day, the cells were washed for 5 min with 1× DPBS two times, followed by treatment with the secondary antibodies, as outlined in Table S3, in 3% BSA in 1× DPBS for 1 h on a rocker at RT. The cells were then washed for 5 min with 0.1% Tween 20 (Millipore Sigma; cat: 655204) in 1× DPBS two times. The cells were then washed for 5 min in 1× DPBS. Coverslips were then dipped into ddH_2_O to remove any residual salt crystals and mounted on microscope slides (Fisher Scientific; cat: 22-034486) using ProLong^TM^ Diamond Antifade Mountant with DAPI (Thermo Fisher; cat: P36962). The transwell membranes were cut out of the inserts before mounting onto microscope slides with an additional sealing layer with a rectangular #1.5 coverglass (Chemglass; cat: 48393-195) mounted on the membranes. The slides were allowed to cure overnight before imaging.

#### Fluorescent Imaging w/“Grid” Confocal Microscope

The slides as prepared above were imaged on an Olympus BX51 microscope connected to a Hamatsu ORCA-ER detector and illuminated with a Prior Lumen 200 source with a Hg lamp (Prior; cat LM200B1-A). Excitation lines and bandpass emission filter (BPF) pairs are as follows—350 nm/DAPI (Semrock; cat: FF02-447/60-25); 405 nm/FITC (Semrock; cat: FF01-524/24-25); 488 nm/TRITC (Semrock; cat: FF01-593/40-25); 568 nm/Cy5 (Semrock; cat: FF01-692/40-25); 350 nm/TRITC—and were used to capture PL from DAPI, AlexaFluor488, AlexaFluor568, AlexaFluor647, and CdSe/CdS QD, respectively. The emission was collected through an infinity-corrected 20x UPlanApo 0.70 NA objective (Olympus). The emission is then passed through an OptiGrid structured illumination element to form a grid confocal image on the detector. For each sample, a z-stack was captured at interval steps of 1 μm and compressed into the extended focus view presented in the representative images used in this manuscript. The exposure time for each channel was optimized and kept the same between each sample.

#### Volocity Image Analysis

The acquired z-stacks were analyzed using the Volocity 3D Image Analysis software (PerkinElmer). A fine noise filter was used on all images before applying a set of measurement protocols. For the bEnd.3 monolayers, a measurement protocol was designed to identify objects above a certain threshold corresponding to immunofluorescent labeled nuclei (λ_ex_ = 350 nm, DAPI BPF), PECAM-1 (λ_ex_ = 405 nm, FITC BPF), CLDN-5 (λ_ex_ = 488 nm, TRITC BPF), and VCAM-1 (λ_ex_ = 568 nm, Cy5 BPF), as well as CdSe/CdS QD emission (λ_ex_ = 350 nm, TRITC BPF). The sum of the measured intensities and the sum of total spatial volume (i.e., voxels) from the objects were then exported for further analysis for the PECAM-1, CLDN-5, and VCAM-1 objects. The total number of detected nuclei and CdSe/CdS objects were also exported. For the neuroglial cultures, a measurement protocol was designed to identify objects above a certain threshold corresponding to immunofluorescent labeled nuclei (λ_ex_ = 350 nm, DAPI BPF), PSD-95 (λ_ex_ = 405 nm, FITC BPF), MAP2 (λ_ex_ = 488 nm, TRITC BPF), and GFAP (λ_ex_ = 568 nm, Cy5 BPF), as well as CdSe/CdS QD emission (λ_ex_ = 350 nm, TRITC BPF). The sum of measured object intensities and spatial volume was extracted for the PSD-95, MAP-2, and GFAP objects. The MAP-2 objects were also further analyzed to extract the prevalence of dendritic beading by setting a cutoff for object volume and threshold for spheroidicity to identify true “beads.” The total number of beads, nuclei, and CdSe/CdS objects were also exported for further analysis.

### Statistical Analysis

All quantitative values were organized and pre-processed in Excel prior to importing the values onto GraphPad Prism 9. Each replicate value was imported. For the rescue experiments of the NVU co-culture experiments, a two-way ANOVA with Holm-Sidak post hoc correction was used. For all other experiments, a one-way ANOVA with Holm-Sidak post hoc correction was used. Statistical significance was defined as an adjusted *p*-value less than 0.05 for all analyses.

## Data Availability

Files associated with the data reported here are accessible at OSF.io (https://osf.io/5q7ye/?view_only=3467ab2052164a75b0144c8f9b19a900).

## Supporting Information Available

MALDI-ToF and NMR spectra to characterize PE:PEG:bis-sulfone synthesis; additional grid confocal fluorescent images; TEM images to characterize COVID-QDs; cell viability resazurin assay of bEnd.3 cells in response to various compounds used in studies; tables denoting the specific primary and secondary antibodies used to label bEnd.3 cultures and primary neuroglia in this study, as well as their relevant dilutions (PDF)
