# Supplementary material for: Quantum Dot Biomimetic for SARS-CoV-2 to Interrogate Blood–Brain Barrier Damage Relevant to NeuroCOVID Brain Inflammation
Source: ACS Appl Nano Mater. 2023 Aug 7;6(16):15094–107. doi: 10.1021/acsanm.3c02719 (PMC10463222; doi:10.1021/acsanm.3c02719)
Supplement: Supplementary file 1 — an3c02719_si_001.pdf [file an3c02719_si_001.pdf]

Supporting Information

A Quantum Dot Biomimetic for SARS-CoV-2 to Interrogate Blood-Brain Barrier Damage Relevant to NeuroCOVID Brain Inflammation

Wesley Chiang[1], Angela Stout[2], Francine Yanchik-Slade[3], Herman Li[2], Niccolo Terrando[6]. Bradley L. Nilsson[3], Harris A. Gelbard*[2,4], Todd D. Krauss*[3,5]

[1]Department of Biochemistry and Biophysics, [2]Center for Neurotherapeutics Discovery and Department of Neurology, [4]Departments of Pediatrics, Neuroscience, and Microbiology and Immunology, University of Rochester Medical Center, Rochester, New York 14642, United States

[3]Department of Chemistry, [5]The Institute of Optics, University of Rochester, Rochester, New York 14627, United States

[6]Department of Anesthesia, Duke University Medical Center, Durham, North Carolina 27710, United States

*Corresponding Authors

Todd Krauss | todd.krauss@rochester.edu | Phone: (585) 275-5093

Harris Gelbard | harris_gelbard@urmc.rochester.edu | Phone: (585) 275-7391

FIGURES 3
Figure S1. MALDI-ToF Spectra for (A) NHS-bis-sulfone (B) DSPE-PEG$_{2k}$-NH$_2$ and (C) PE:PEG:bis-sulfone. 3
Figure S2. NMR spectra for PE:PEG:bis-sulfone (A) 1H (B) 13C.. 4
Figure S3. Example of typical absorption and photoluminescence spectra of CdSe/CdS QDs synthesized and used in this study. 5
Figure S4. Optical spectra corresponding to TEM and EDX analysis of QD-micelle solutions to confirm removal of empty and smaller QD-micelle species to result in a high-purity QD-micelle solution containing larger QD-micelles. The reported concentrations on the plots reflect the concentration of CdSe/CdS QDs, estimated via the band-edge absorption peak, mapped onto the CdSe extinction coefficient reported in Yu *et al.*[1] 6
Figure S5. Comparative example of QD-micelle samples constructed with insufficient (left) and sufficient (right) excess of PE:PEG:bis-sulfone monomers during initial encapsulation step. 8
Figure S6. Treatment of bEnd.3 monolayers with either 1nM QD-micelles or 1nM COVID-QDs with 10nM soluble hACE2.. 9
Figure S7. Dose-response of bEnd.3 monolayers to a dilution series of 1nM, 3nM, and 10nM COVID-QD treatments to show saturation of functional dysregulation in monolayers. 9
Figure S8. Treatment of *in vitro* co-culture model system of static NVU with tight junction disrupting peptide derived from extracellular loop of Claudin-1. 10
Figure S9. Resazurin metabolism viability assay of bEnd.3 cells in response to various independent reagents used in this study. Reported values are mean $\pm$ SEM and all were found to be not significant (adjusted p $\geq$ 0.05). n = 7 cultures pooled from two passages. 11
Figure S10. Rescue of bEnd.3 monolayers in transwell co-culture with neurons and astrocytes by pre-treating with 200 nM URMC-099 before treatment with either 10nM of Spike protein or 1 nM of COVID-QDs. Scale bars = 15 μm. Nuclei stained by DAPI (deep blue) are shown in all panels. 12
Figure S11. Rescue of bEnd.3 monolayers in transwell co-culture with neurons and astrocytes by co-treating 10 nM of soluble hACE2 with either 10 nM of Spike protein or 1nM of COVID-QDs. This leads to no formation of intracellular clusters of CLDN-5 nor induction of significant VCAM-1 expression. Scale bars = 15 μm. Nuclei stained by DAPI (deep blue) are shown in all panels...13
Figure S12. Small molecule rescue of neuron and astrocyte health by either 200 nM URMC-099 pre-treatment or 10 nM soluble hACE2 co-treatment with 10nM Spike protein. Scale bars = 15μm. Nuclei stained by DAPI (deep blue) are shown in all panels. 14
Figure S13. Small molecule rescue of neuron and astrocyte health by either 200 nM URMC-099 pre-treatment or 10 nM soluble hACE2 co-treatment with 1nM COVID-QDs. Scale bars = 15μm. Nuclei stained by DAPI (deep blue) are shown in all panels. 15
TABLES 16
Table S1. Parameters for A280 calculation to determine fraction of spike protein in eluted unbound fraction (n = 5). 16
Table S2. Selection of primary antibodies and dilutions used for immunofluorescent staining 16
Table S3. Selection of secondary antibodies used for immunofluorescent staining 16

FIGURES

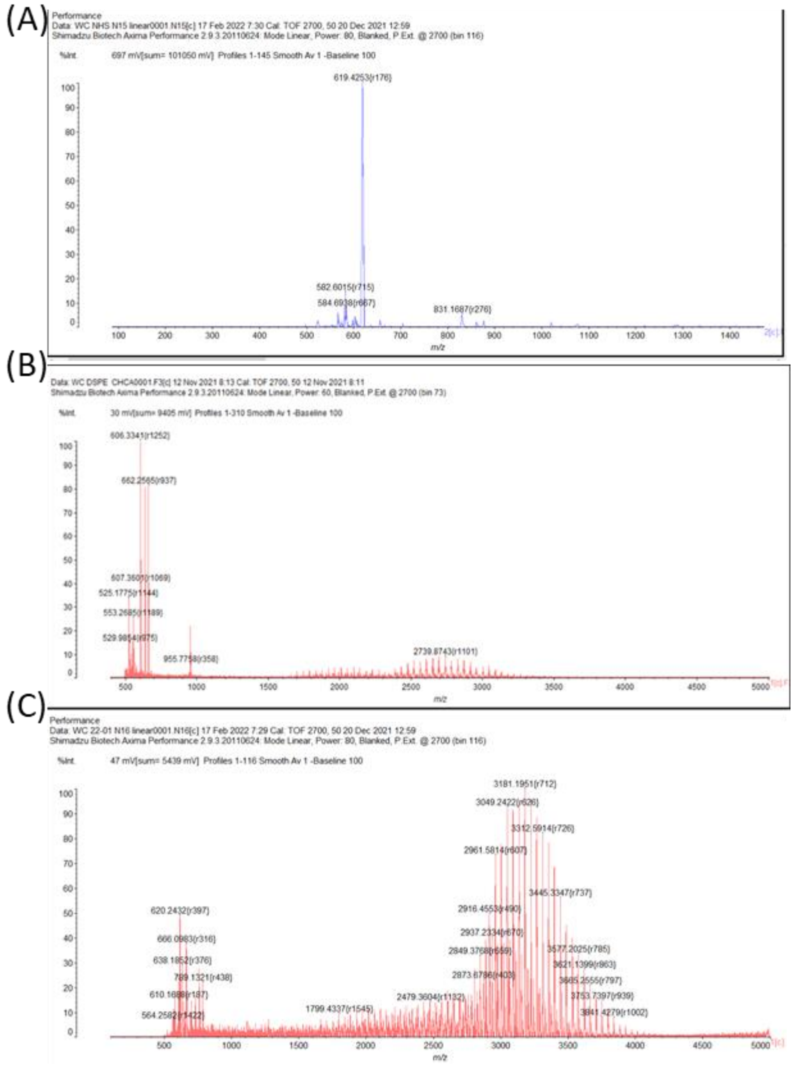

Figure S1. MALDI-ToF Spectra for (A) NHS-bis-sulfone (B) DSPE-PEG_{2k}-NH_2 and (C) PE:PEG:bis-sulfone.

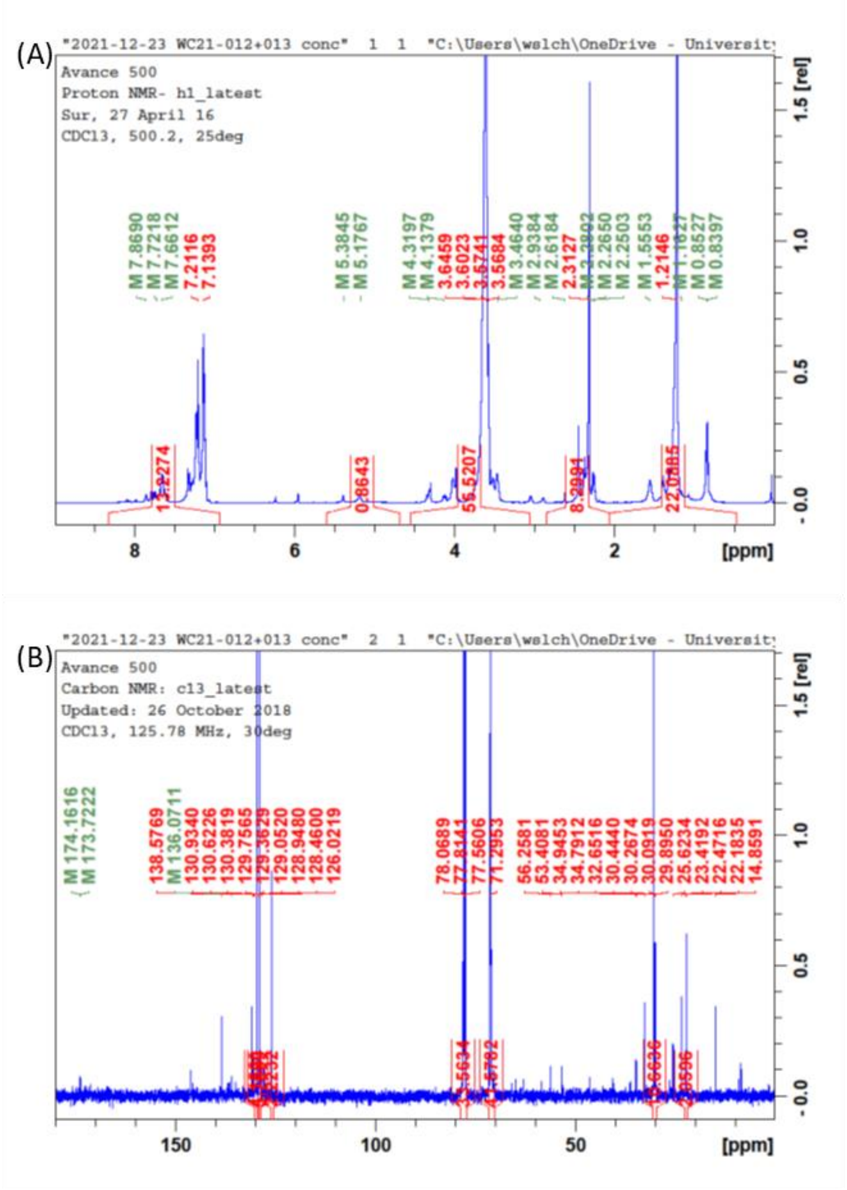

Figure S2. NMR spectra for PE:PEG:bis-sulfone (A) 1H (B) 13C.

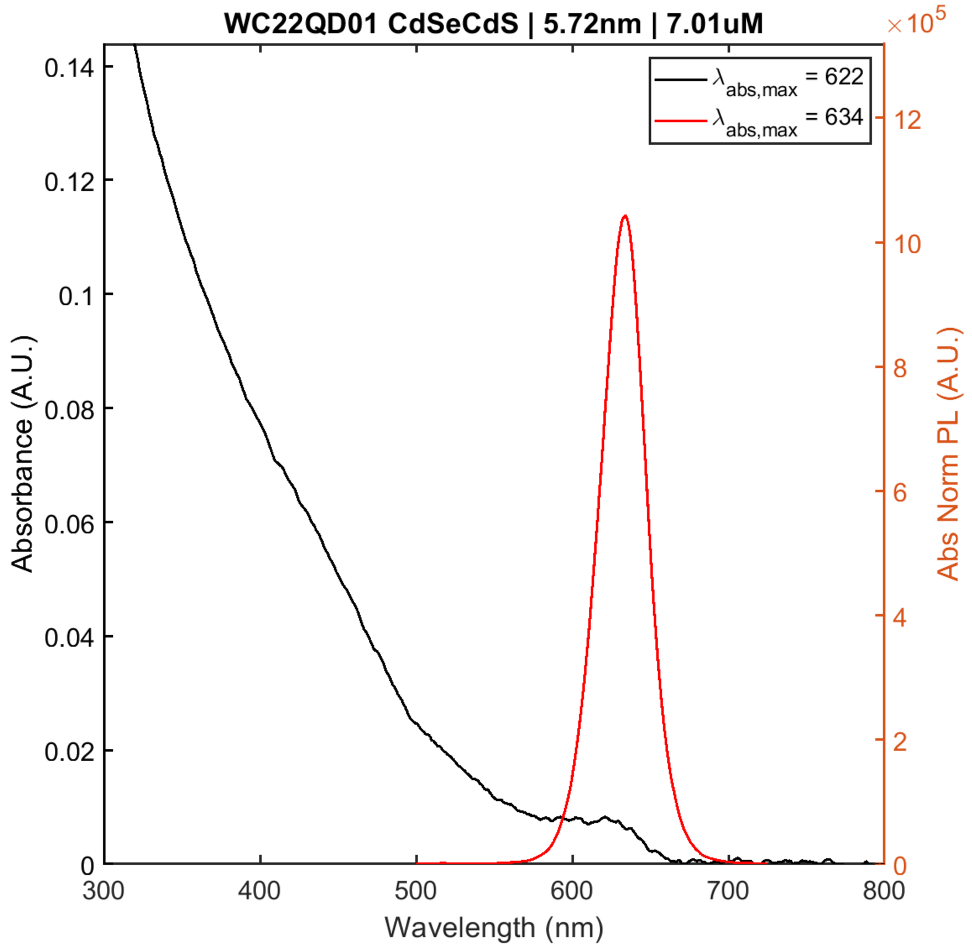

Figure S3. Example of typical absorption and photoluminescence spectra of CdSe/CdS QDs synthesized and used in this study.

Measurements are taken on aliquots that are typically diluted at least 2 orders of magnitude; this dilution factor is used to generate a final stock concentration, as reported in the title of the figure.

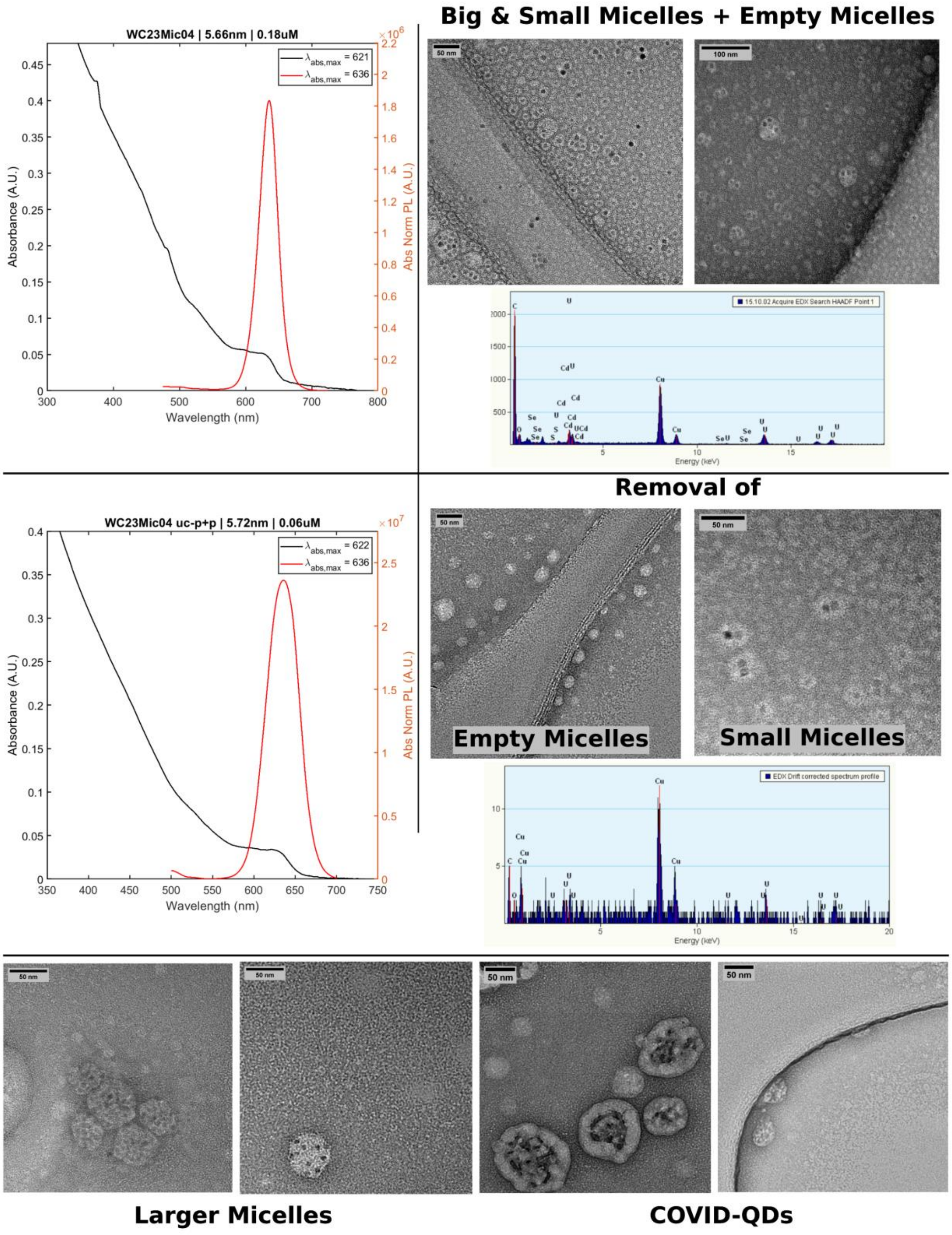

Figure S4. Optical spectra corresponding to TEM and EDX analysis of QD-micelle solutions to confirm removal of empty and smaller QD-micelle species to result in a high-purity QD-micelle solution containing larger QD-micelles. The reported concentrations on the plots reflect the concentration of CdSe/CdS QDs, estimated

via the band-edge absorption peak, mapped onto the CdSe extinction coefficient reported in Yu *et al.*[1]

First row: The presence of empty micelles is evident via the slanted shoulder at higher wavelengths in the black absorbance spectra. This is corroborated by the presence of a mixed species of micelles in the two TEM images; these show empty micelles containing no additional electron dense particle in the interior, as well as micelles of different sizes with QDs in them.

Second row: The shoulder in the black absorbance spectra is attenuated after ultracentrifugation, indicating the removal of empty micelles. The empty micelles are contained in the supernatant after ultracentrifugation; this was collected and spotted for TEM to show empty micelles in the left TEM image. Another round of purification was performed to separate large and small micelles, the latter of which was spotted for TEM and shown in on the right on this row.

Third row: The size pure large QD-micelle species (left two TEM images) were then conjugated with 20x molar of spike proteins to produce the COVID-QD TEM images shown on the right, both at ≤10 nM concentrations of micellar particles. The concentration of the large QD-micelle and resultant COVID-QD solutions were approximated using at average of 8 CdSe/CdS QDs per micelle, as evident by the TEM images, to then adjust the measured concentration of CdSe/CdS in the micelle solution to relative concentration of QD-micelles.

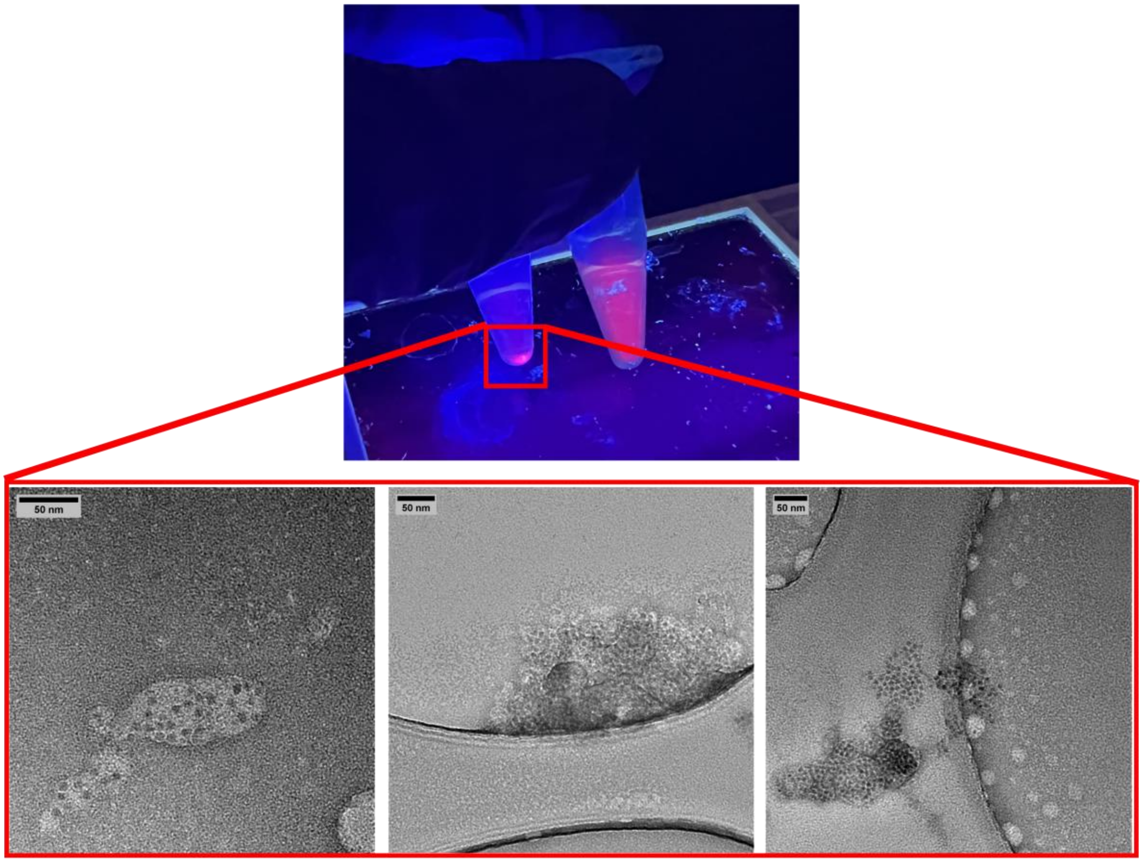

Figure S5. Comparative example of QD-micelle samples constructed with insufficient (left) and sufficient (right) excess of PE:PEG:bis-sulfone monomers during initial encapsulation step.

This results in differential stability of micellar solutions after successive rounds of purification and dilution to final working concentrations. The unstable QD-micelles at ≤10 nM result in CdSe/CdS QDs that pellet out of aqueous solution after extended storage, that can be temporarily resuspended in solution, but TEM analysis of these resuspended solutions exhibit asymmetrical encapsulation of QDs and QD-polymer aggregates that do not resemble the micelles from stable QD-micelle and COVID-QD solutions shown in Figure S3.

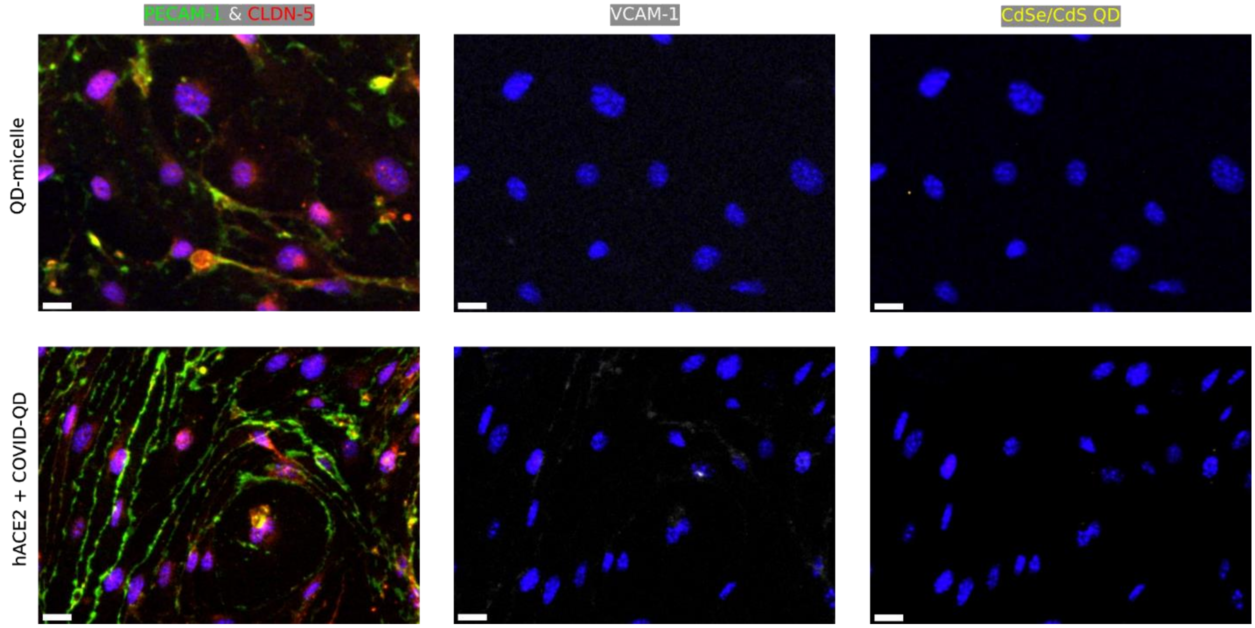

Figure S6. Treatment of bEnd.3 monolayers with either 1 nM QD-micelles or 1 nM COVID-QDs with 10 nM soluble hACE2.

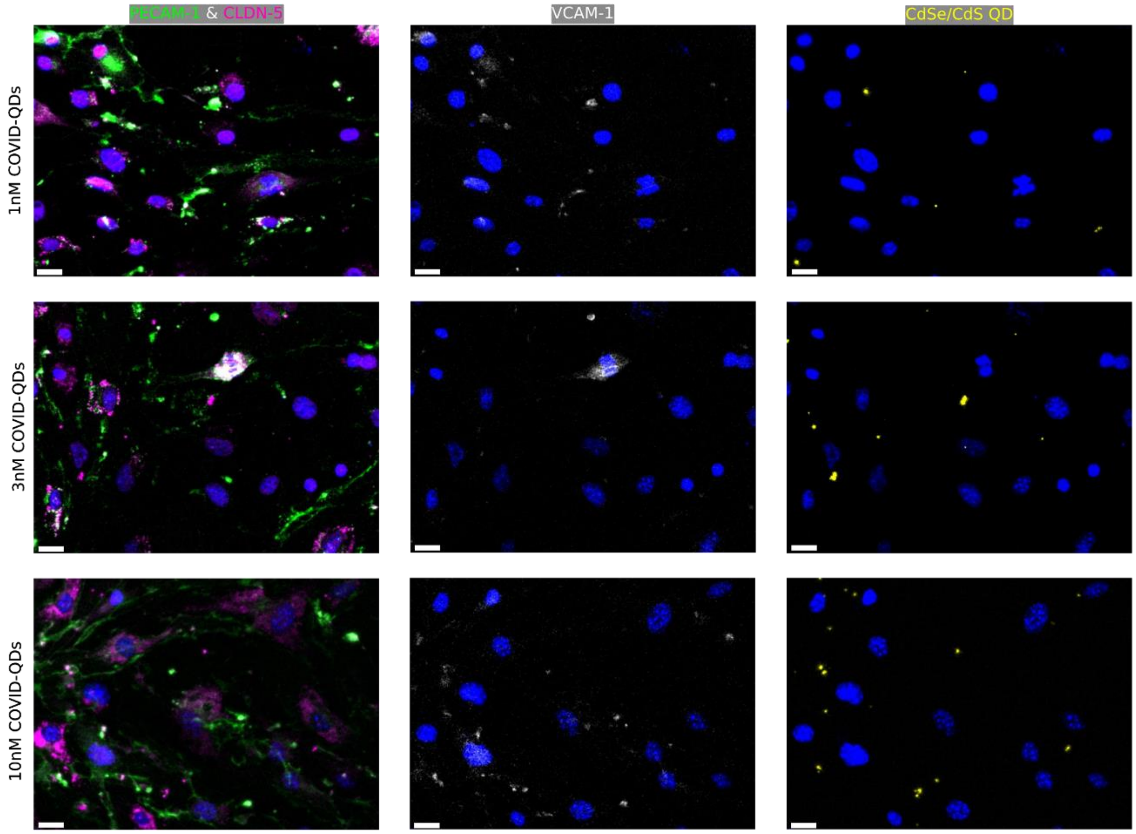

Figure S7. Dose-response of bEnd.3 monolayers to a dilution series of 1 nM, 3 nM, and 10 nM COVID-QD treatments to show saturation of functional dysregulation in monolayers.

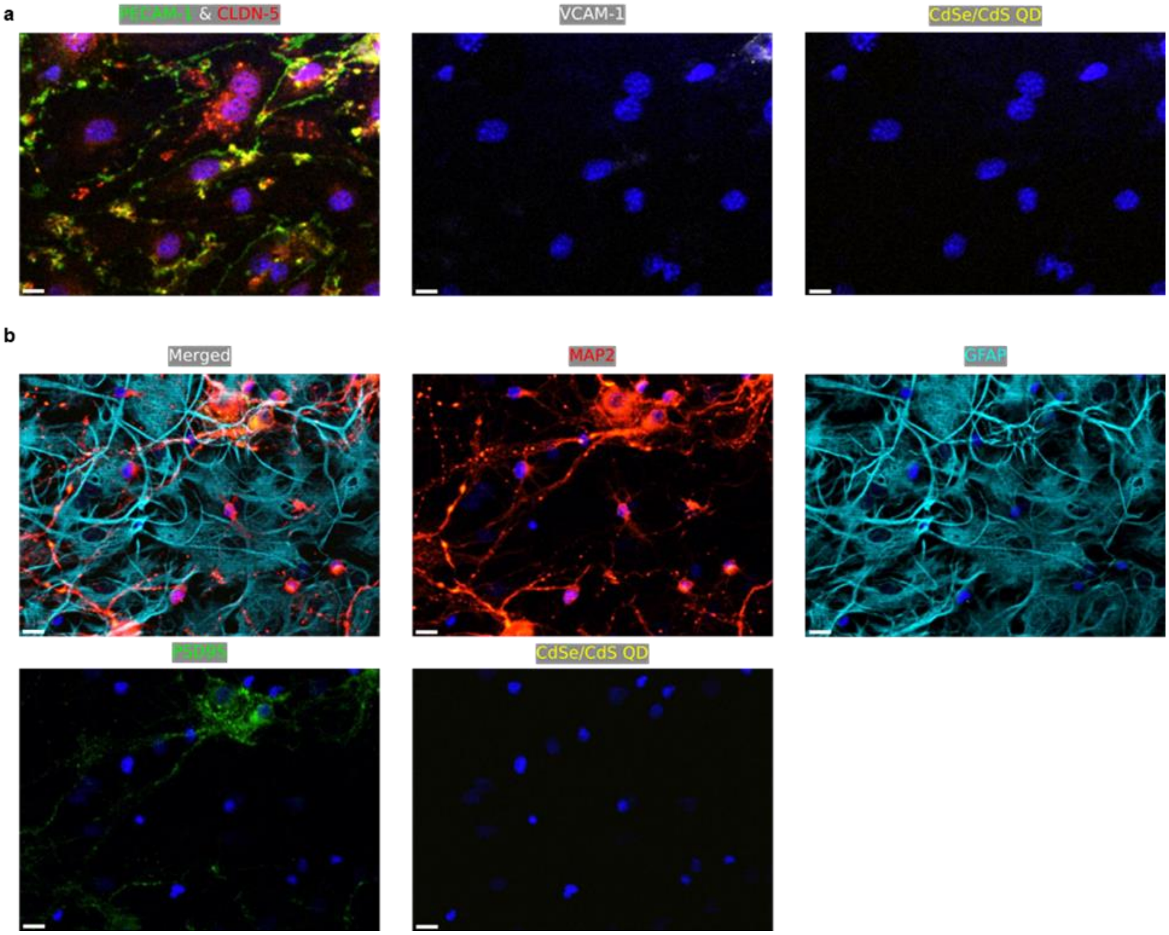

Figure S8. Treatment of *in vitro* co-culture model system of static NVU with tight junction disrupting peptide derived from extracellular loop of Claudin-1.

a, bEnd.3 monolayers with a tight junction disrupting peptide (TJDP) derived from the extracellular loop of Claudin-1 that leads to dysregulation of CLDN-5 localization away from membrane and into intracellular clusters without induction of significant inflammation, noted by VCAM-1 expression. **b,** Subsequent neuroinflammatory effects in neuroglia co-cultured under bEnd.3 monolayers in response to TJDP that leads to formation of dendritic beading, or puncta in MAP2, and astrogliosis, as represented by increased density of GFAP. Interestingly, while the TJDP treatment group did not induce inflammation in bEnd.3 monolayers we found a significant increase in GFAP density, indicating potential astrogliosis in this treatment group. We presume that the observed astrocytosis is a secondary response to significantly greater leakage in the TJDP treatment groups compared to the S and COVID-QD treatment groups[2-4].

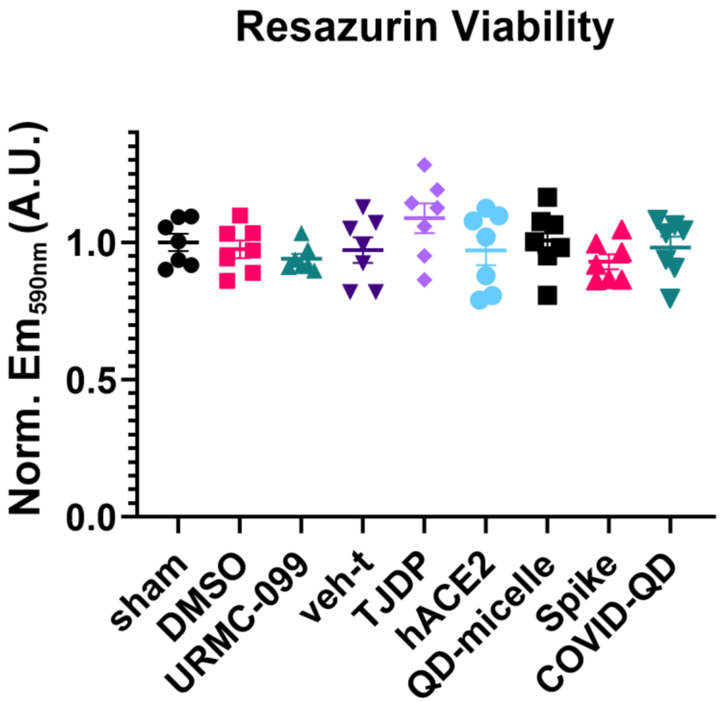

Figure S9. Resazurin metabolism viability assay of bEnd.3 cells in response to various independent reagents used in this study. Reported values are mean ± SEM and all were found to be not significant (adjusted $p \geq 0.05$). $n = 7$ cultures pooled from two passages.

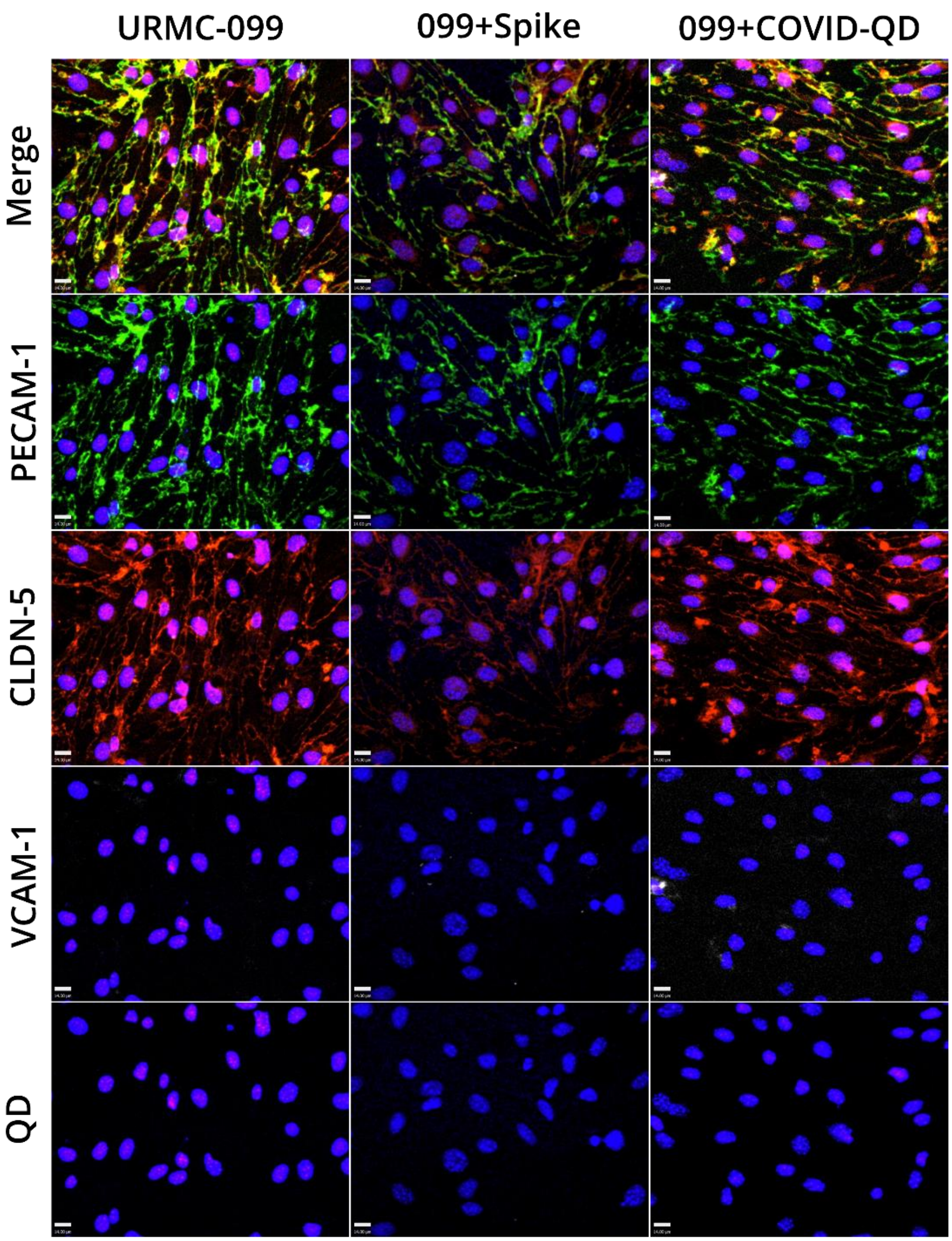

Figure S10. Rescue of bEnd.3 monolayers in transwell co-culture with neurons and astrocytes by pre-treating with 200 nM URMC-099 before treatment with either 10 nM of Spike protein or 1 nM of COVID-QDs. Scale bars = 15 µm. Nuclei stained by DAPI (deep blue) are shown in all panels.

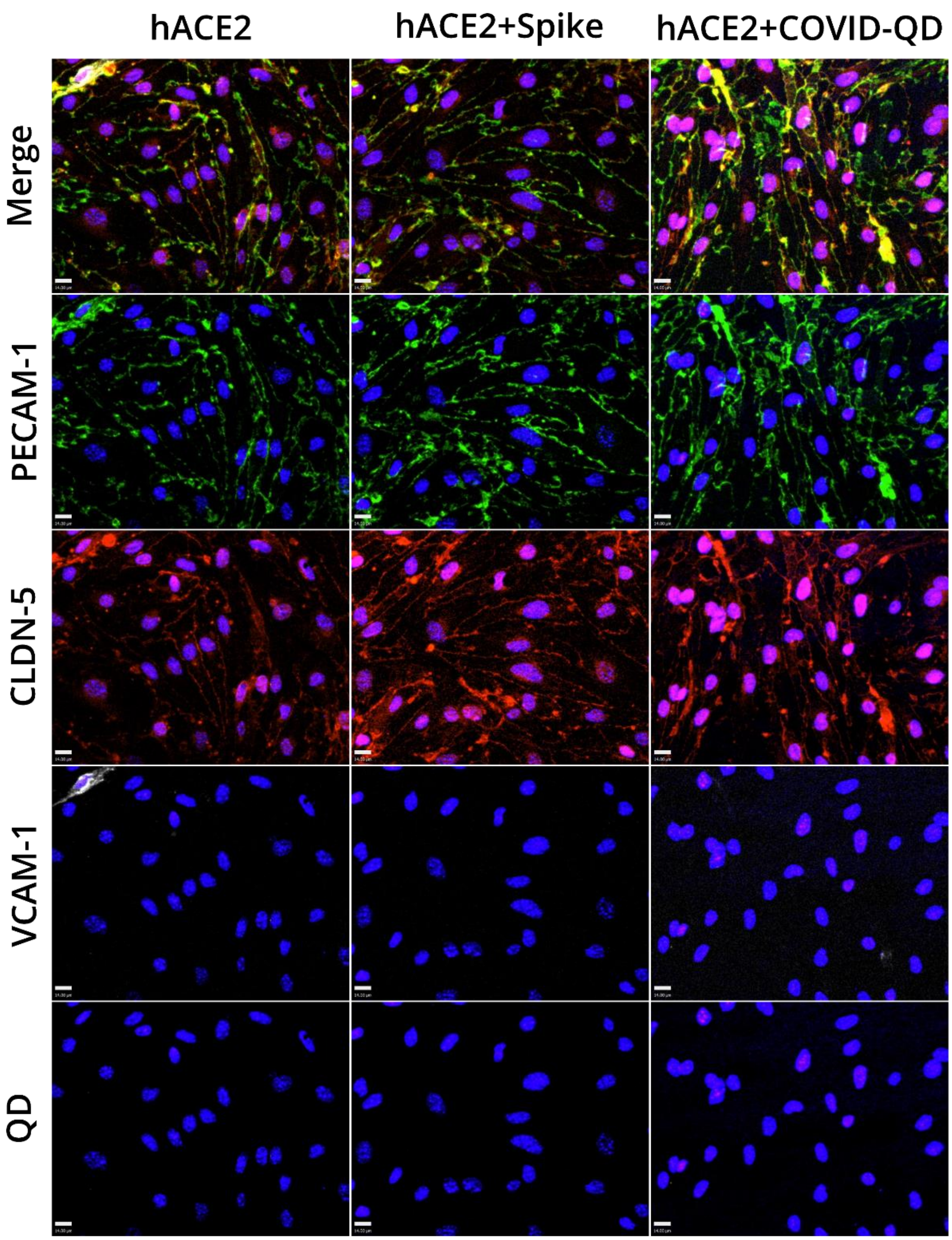

Figure S11. Rescue of bEnd.3 monolayers in transwell co-culture with neurons and astrocytes by co-treating 10 nM of soluble hACE2 with either 10 nM of Spike protein or 1 nM of COVID-QDs. This leads to no formation of intracellular clusters of CLDN-5 nor induction of significant VCAM-1 expression. Scale bars = 15 µm. Nuclei stained by DAPI (deep blue) are shown in all panels.

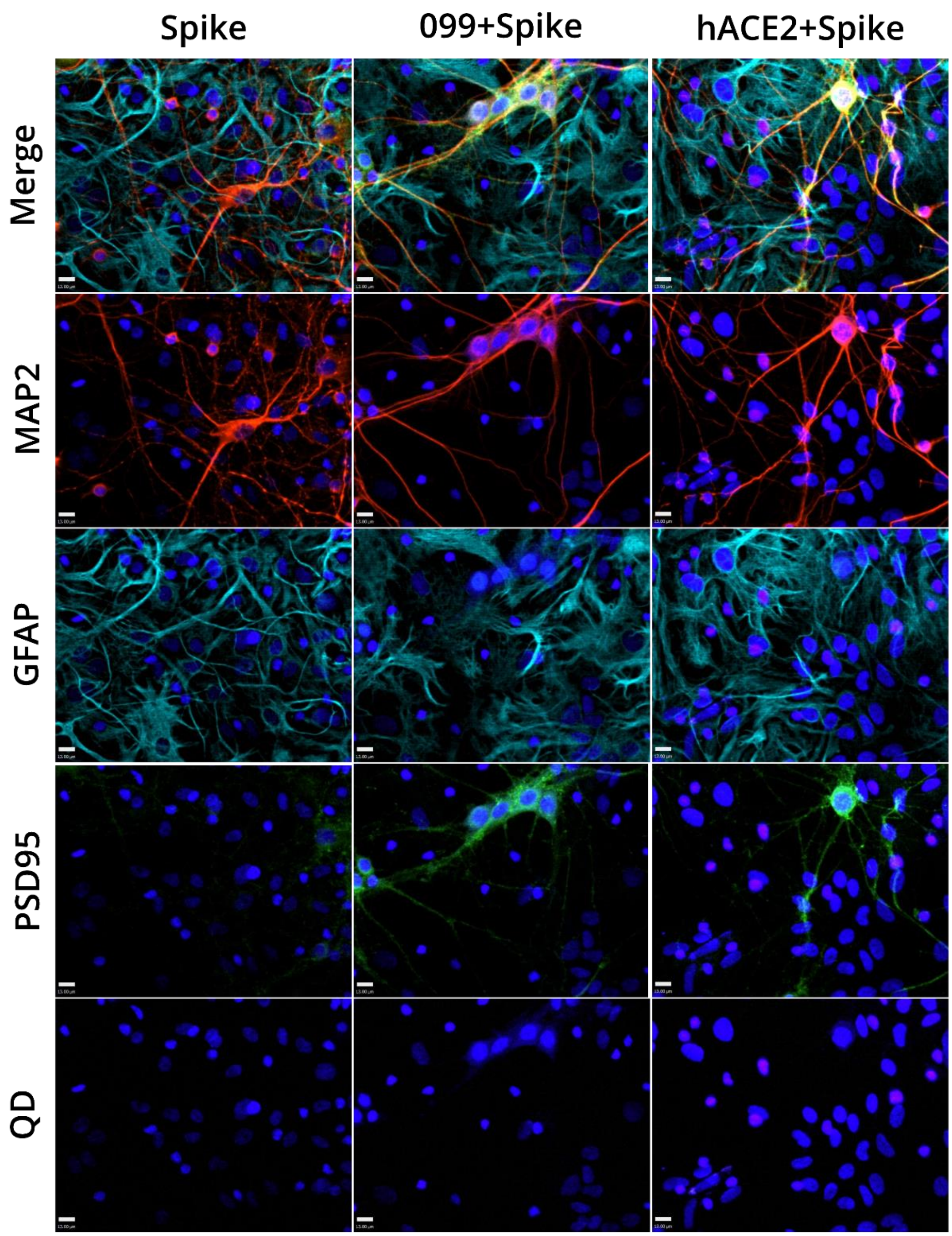

Figure S12. Small molecule rescue of neuron and astrocyte health by either 200 nM URMC-099 pre-treatment or 10 nM soluble hACE2 co-treatment with 10 nM Spike protein. Scale bars = 15 μm. Nuclei stained by DAPI (deep blue) are shown in all panels.

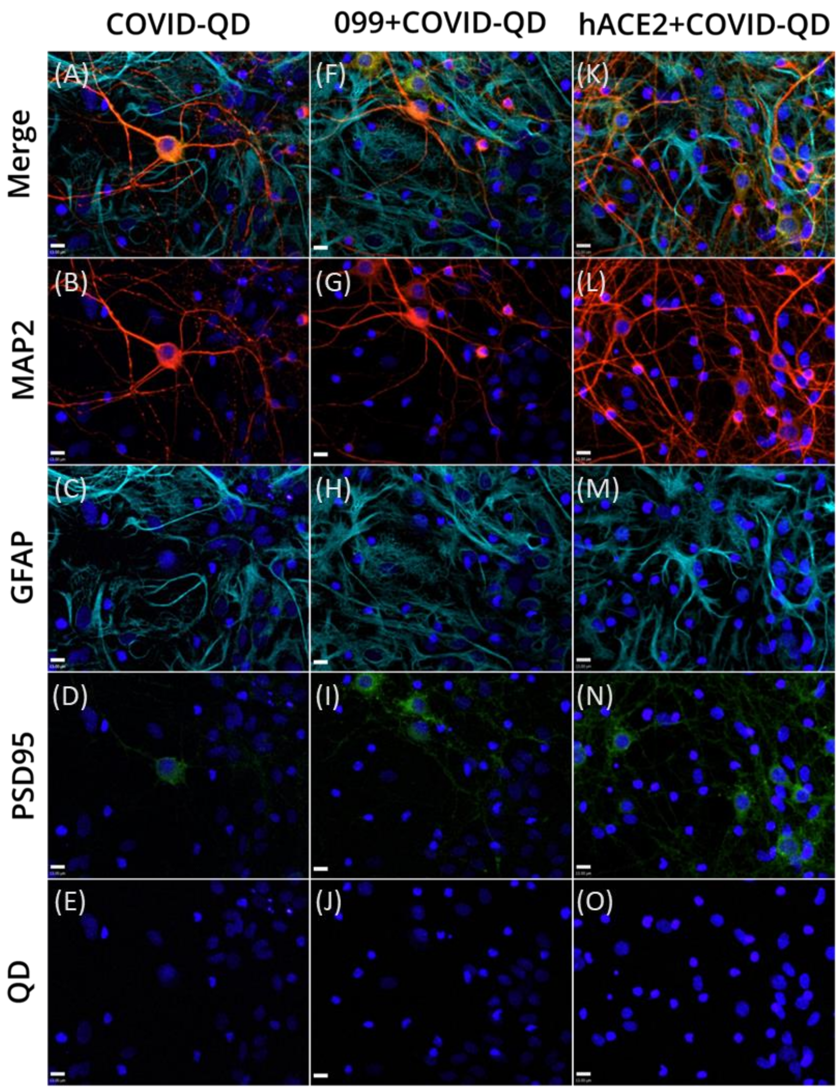

Figure S13. Small molecule rescue of neuron and astrocyte health by either 200 nM URMC-099 pre-treatment or 10 nM soluble hACE2 co-treatment with 1 nM COVID-QDs. Scale bars = 15 µm. Nuclei stained by DAPI (deep blue) are shown in all panels. (A-E) COVID-QD treated co-cultures result in the observation of beading (B), astrogliosis (C), and loss of synaptic densities (D) that are not associated with COVID-QD migration across brain endothelium (E). This is rectified by both treatment with URMC-099 (F-J) and hACE2 (K-O), where the neurons exhibit less discontinuities (G & L) and recovered synaptic densities (I & N), while the astrocytes appear less activated (H & M).

TABLES

**Table S1.** Parameters for A280 calculation to determine fraction of spike protein in eluted unbound fraction (n = 5).		
MW	ϵ_{calc}	$\%Spike_{unbound}$
135kDa	166,860	12.5 ± 1.2

**Table S2.** Selection of primary antibodies and dilutions used for immunofluorescent staining					
Culture	***Host***	***Target***	***Dilution***	***Source***	***Catalog #***
bEnd.3	Hamster	PECAM-1	1:200	ThermoFisher	MA3105
	Rabbit	CLDN-5	1:200	ThermoFisher	PA5-99415
	Rat	VCAM-1	1:200	BD Biosciences	553330
neurons	Mouse	PSD95	1:200	NeuroMab	75-028
	Rabbit	MAP2	1:150	Cell Signaling	4542
astrocytes	Chicken	GFAP	1:250	Neuromics	CH22102

**Table S3.** Selection of secondary antibodies used for immunofluorescent staining					
Host	***Reactivity***	***Fluor***	***Dilution***	***Source***	***Catalog***
Goat	Hamster	Alexa 488	1:750	Jackson ImmunoResearch	127-545-099
	Rabbit	Alexa 568		ThermoFisher	A-11036
	Rat	Alexa 647		ThermoFisher	A-21247
	Mouse	Alex 488		ThermoFisher	A-11006
	Chicken	Alexa 647		ThermoFisher	A-21449

REFERENCES

(1) Yu, W. W.; Qu, L.; Guo, W.; Peng, X. Experimental Determination of the Extinction Coefficient of CdTe, CdSe, and CdS Nanocrystals. *Chem. Mater.* **2003**, *15* (14), 2854-2860. DOI: 10.1021/cm034081k.

(2) Horng, S.; Therattil, A.; Moyon, S.; Gordon, A.; Kim, K.; Argaw, A. T.; Hara, Y.; Mariani, J. N.; Sawai, S.; Flodby, P.; Crandall, E. D.; Borok, Z.; Sofroniew, M. V.; Chapouly, C.; John, G. R. Astrocytic tight junctions control inflammatory CNS lesion pathogenesis. *J. Clin. Invest.* **2017**, *127* (8), 3136-3151. DOI: 10.1172/jci91301.

(3) Morizawa, Y. M.; Hirayama, Y.; Ohno, N.; Shibata, S.; Shigetomi, E.; Sui, Y.; Nabekura, J.; Sato, K.; Okajima, F.; Takebayashi, H.; Okano, H.; Koizumi, S. Reactive astrocytes function as phagocytes after brain ischemia via ABCA1-mediated pathway. *Nat. Commun.* **2017**, *8* (1). DOI: 10.1038/s41467-017-00037-1.

(4) Wu, Y.; Wu, H.; Zeng, J.; Pluimer, B.; Dong, S.; Xie, X.; Guo, X.; Ge, T.; Liang, X.; Feng, S.; Yan, Y.; Chen, J.-F.; Sta Maria, N.; Ma, Q.; Gomez-Pinilla, F.; Zhao, Z. Mild traumatic brain injury induces microvascular injury and accelerates Alzheimer-like pathogenesis in mice. *Acta Neuropathol. Commun.* **2021**, *9* (1). DOI: 10.1186/s40478-021-01178-7.
